# Identify the Prognostic and Immune Profile of VSIR in the Tumor Microenvironment: A Pan-Cancer Analysis

**DOI:** 10.3389/fcell.2022.821649

**Published:** 2022-04-12

**Authors:** Yuanyuan Liu, Jingwei Zhang, Zeyu Wang, Xun Zhang, Ziyu Dai, Wantao Wu, Nan Zhang, Zaoqu Liu, Jian Zhang, Peng Luo, Zhipeng Wen, Jing Yu, Hao Zhang, Tubao Yang, Quan Cheng

**Affiliations:** ^1^ Department of Epidemiology and Health Statistics, Xiangya School of Public Health, Central South University, Changsha, China; ^2^ National Clinical Research Center for Geriatric Disorders, Changsha, China; ^3^ Department of Neurosurgery, Xiangya Hospital, Central South University, Changsha, China; ^4^ Department of Oncology, Xiangya Hospital, Central South University, Changsha, China; ^5^ One-third Lab, College of Bioinformatics Science and Technology, Harbin Medical University, Harbin, China; ^6^ Department of Interventional Radiology, The First Affiliated Hospital of Zhengzhou University, Zhengzhou, China; ^7^ Department of Oncology, Zhujiang Hospital, Southern Medical University, Guangzhou, China; ^8^ Department of Pharmacy, The Affiliated Hospital of Guizhou Medical University, Guizhou Medical University, Guiyang, China; ^9^ Department of Clinical Pharmacology, Xiangya Hospital, Central South University, Changsha, China; ^10^ Clinical Diagnosis and Therapy Center for Glioma of Xiangya Hospital, Central South University, Changsha, China

**Keywords:** VSIR, immunotherapy, macrophages, t cells, tumor microenvironment

## Abstract

VSIR is a critical immunomodulatory receptor that inhibits T cell effector function and maintains peripheral tolerance. However, the mechanism by which VSIR participates in tumor immunity in the pan-cancer tumor microenvironment remains unclear. This study systematically explored the prognostic and immune profile of VSIR in the tumor microenvironment of 33 cancers. We compared the expression patterns and molecular features of VSIR in the normal and cancer samples both from the public databases and tumor chips. VSIR level was significantly related to patients’ prognosis and could be a promising predictor in many tumor types, such as GBM, KIRC, SKCM, READ, and PRAD. Elevated VSIR was closely correlated with infiltrated inflammatory cells, neoantigens expression, MSI, TMB, and classical immune checkpoints in the tumor microenvironment. Enrichment signaling pathways analysis indicated VSIR was involved in several immune-related pathways such as activation, proliferation, and migration of fibroblast, T cell, mast cell, macrophages, and foam cell. In addition, VSIR was found to widely express on cancer cells, fibroblasts, macrophages, and T cells in many tumor types based on the single-cell sequencing analysis and co-express with M2 macrophage markers CD68, CD163 based on the immunofluorescence staining. Finally, we predicted the sensitive drugs targeting VSIR and the immunotherapeutic value of VSIR. In sum, VSIR levels strongly correlated with the clinical outcome and tumor immunity in multiple cancer types. Therefore, therapeutic strategies targeting VSIR in the tumor microenvironment may be valuable tools for cancer immunotherapy.

## Introduction

The emergence of new diagnostic methods and therapeutic strategies over the past few decades has significantly improved the quality of life and survival in patients with advanced cancers (Davis et al., 2017; Arnold et al., 2019). The 5 year relative survival was more than 60% for all cancers combined based on the global cancer epidemiological survey calculated by the National Cancer Control Indicators. Cancer mortality has steadily declined from its peak in 1991–2018, with an overall decrease of more than 30% (Siegel et al., 2020). However, the prognosis in patients with malignant cancers remains far from satisfactory. For example, the 5-year survival rate was high for cancer of the prostate (95%), melanoma (92%), and female breast cancer (91%). In comparison, it was low for cancers of the pancreas (11%), lung cancer (19%), liver cancer (20%), and central nervous system (CNS) tumors (22%) (Bach et al., 2020). In addition, the number of new diagnostic cancer cases and cancer-related deaths are still high. For example, a recent study indicated that there would be more than 1.8 million new cancer cases and nearly 600,000 million cancer deaths expected in the United States in 2021 (Siegel et al., 2021).

Cancer cells are located in a complex and dynamic ecosystem called the tumor microenvironment (TME). TME consists of cancer cells, non-malignant cells, blood vessels, extracellular matrix (ECM), various cytokines, and growth factors (Baghban et al., 2020; Yang et al., 2022). Both factors decided the tumor formation and development, including genetic variation and the rearrangement of the components of the TME through interconnections (Jahanban-Esfahlan et al., 2017). Increasing evidence revealed that tumor immunity regulated by infiltrated inflammatory cells in the TME plays an essential role in the whole process of tumorigenesis and metastasis (Vitale et al., 2019; Lim et al., 2020; Sahai et al., 2020; Zhang et al., 2021c; Ping et al., 2021). Significantly, the specific antibodies that block immune checkpoints secreted by immune infiltrates have become a novel weapon in fighting cancer and have achieved good results in preclinical studies (Dyck and Mills, 2017; Zhang et al., 2018; He and Xu, 2020). For example, programmed death 1 (PD-1) and the cytotoxic T-lymphocyte-associated antigen 4 (CTLA-4) are the top two crucial immune checkpoints that are mainly produced by activated T cells in the TME (Gao et al., 2020; Zhang et al., 2021a). Both play an essential role in negatively mediating T cell immune function during different stages of T-cell activation in tumor immunity. The regulation of PD-1 and CTLA-4 levels in the TME has significantly affected the immunotherapy response and therapeutic effect (Qin et al., 2019). Therefore, discovering and understanding the in-depth mechanisms of new immune checkpoints could prolong the survival time of cancer patients and improve their quality of life.

V-Set Immunoregulatory Receptor (VSIR), also known as VISTA, C10orf54, PD-1H, B7H5, GI24, PP2135, SISP1, is a type 1 transmembrane protein that comes from the immunoglobulin superfamily (Yoon et al., 2015). Two potential protein kinase C binding sites and proline docking residues construct the VSIR cytoplasmic tail domain, indicating that VSIR can act as a receptor or ligand in cellular progress (Le Mercier et al., 2015). Multiple cell types can express VSIR, such as cancer cells, neutrophils, monocytes, macrophages, dendritic cells (D.C.s), and T cells in humans and mice (Flies et al., 2011; Lines et al., 2014). Previous studies have found that VSIR is widely involved in various physiological and pathological processes, including regulating peripheral tolerance, inducing T cell activation and differentiation, and mediating tumor immunity (ElTanbouly et al., 2019; Hosseinkhani et al., 2021). Thus, growing evidence indicated that VSIR might be a promising target for tumor immunotherapeutic intervention in the TME.

To date, most evidence has focused on the suppressive effect role of VSIR in the immune system and the ability to regulate antitumor immunity. However, the mechanisms of VSIR in tumor immunity regulation remain controversial. There are also compelling studies that do not support the value of VSIR as an immunotherapy target. VSIR plays both negative and positive roles in tumor immunity. Upregulated VSIR on the tumor-infiltrating myeloid cells has been found to promote tumor growth through suppressing T cell immunity (Mulati et al., 2019). The specific antibody that neutralizes VSIR can effectively suppress tumor growth in mouse models (Le Mercier et al., 2014). A previous study indicated that higher VSIR levels are related to better clinical prognosis in epithelioid mesothelioma cancer (Muller et al., 2020), non-small-cell lung cancer (Villarroel-Espindola et al., 2018), and esophageal adenocarcinoma (Villarroel-Espindola et al., 2018). However, Lawrence F. Kuklinski et al. found that increased VSIR was associated with unfavorable DSS in primary cutaneous melanoma (Kuklinski et al., 2018).

Therefore, in this study, we systematically investigated the predictive value of VSIR in pan-cancer using large-scale RNA-sequencing (RNA-seq) data from the public databases. We also explored the relationship between VSIR and immune infiltration in different cancer TME. Meanwhile, the co-expression of VSIR on infiltrated inflammatory cells was studied from both online databases and tumor chip data. In addition, we evaluated the immunotherapy value of VSIR in pan-cancer from various immunotherapy cohorts. Finally, we predicted many sensitive small molecule drugs based on VSIR expression from the public databases.

## Materials and Methods

### Data Collection and Collation

We obtained the RNA-seq data from the TCGA (pan-cancer) (https://portal.gdc.cancer.gov/), GETX (normal control) (https://gtexportal.org/home/), and CCLE (cancer cell lines) (https://sites.broadinstitute.org/ccle/) datasets. In addition, the single-cell sequencing data were downloaded from the GEO database (https://www.ncbi.nlm.nih.gov/geo/), including invasive breast carcinoma (BRCA, GSE75688, and GSE118389), cholangiocarcinoma (CHOL, GSE125449), colon adenocarcinoma (COAD, GSE81861), head and neck squamous cell carcinoma (HNSC, GSE103322), liver hepatocellular carcinoma (LIHC, GSE125449), bladder cancer (BLCA, GSE145137), kidney renal clear cell carcinoma (KIRC, GSE121636 and GSE171306), ovarian serous cystadenocarcinoma (OV, GSE118828), prostate adenocarcinoma (PRAD, GSE137829), skin cutaneous melanoma (SKCM, GSE72056), stomach adenocarcinoma (STAD, GSE183904). Besides, the single-cell sequencing data of glioblastoma multiforme (GBM, SCP50, and SCP393) was downloaded from the Single Cell Portal platform (http://singlecell.broadinstitute.org); the single-cell sequencing dataset of LUAD was downloaded from the BioProject (PRJNA591860).

### Identification of Relevant Features

The mutant genetic aspects of VSIR were analyzed by the GSCA(Liu et al., 2018) (http://bioinfo.life.hust.edu.cn/GSCA/) and CBIOPORTAL (https://www.cbioportal.org/) databases. The Kaplan-Meier (KM) analysis with log-rank test was used to calculate the disease-specific survival (DSS) and overall survival (OS) of pan-cancer patients (Lanczky and Gyorffy, 2021). We explored the immune characteristics of VSIR through TIMER 2.0 (Li et al., 2017) (https://cistrome.shinyapps.io/timer/) and R package (immunedeconv). The gene set variation analysis (GSVA) based on Gene Ontology (GO) terms and the gene set enrichment analysis (GSEA) based on Kyoto Encyclopedia of Genes and Genomes (KEGG) and HALLMARK gene sets were used to identify the enriched signaling pathways related to VSIR. The TIDE (http://tide.dfci.harvard.edu) and TISMO (http://tismo.cistrome.org) websites were selected to evaluate the immunotherapy value of VSIR. The involvement of VSIR in diseases and aid systematic drug target identification and prioritization was identified from the OPEN TARGET platform (https://www.targetvalidation.org/). The GSCA database was used to predict sensitive drugs based on VSIR expression. The GSCA data contains over 750 small-molecule drugs from GDSC and CTRP for 10,000 genomic data across 33 cancer types.

### Single-Cell Sequencing Analysis

The single-cell sequencing data of BRCA was integrated using the R package Seurat. Quality control was conducted by the R package (Seurat) (Butler et al., 2018). Principal component analysis (PCA) was used for dimension reduction. FindClusters function was used to cluster cells. The R package, infercnv, and copykat were applied to explore the identification of tumor cells. The UMAP function was used for the visualization of dimensionality reduction. Vlnplot, Dimplot, and Featureplot were used for visualizing VSIR expression.

### Immunofluorescence Staining

We obtained the tissue microarray from the Outdo Biotech company (HOrg-C110PT-01, total number of cases: 69 cases, total points: 110 points, Shanghai, China). The tissue microarray was approved by the Ethics Committee. Each tumor/normal tissue has three to eight cores (diameter 1.5 mm). The primary Abs were VSIR (Rabbit, 1:200, Proteintech, China), CD68 (Rabbit, 1:3000, AiFang biological, China), CD163 (Rabbit, 1:3000, Proteintech, China), CD8 (Mouse, 1:3000, Proteintech, China). The secondary antibody was horseradish peroxidase-conjugated secondary antibody incubation (GB23301, GB23303, Servicebio, China), and the tyramide signal amplification was TSA (FITC-TSA, CY3-TSA, 594-TSA, and 647-TSA (Servicebio, China)). Multispectral images were analyzed, and positive cells were quantified at single-cell levels by Caseviewer (CV 2.3, CV 2.0) and Pannoramic viewer (PV 1.15.3) image analysis software. Negative control procedures included the omission of the primary antibody.

### Statistical Analysis

We calculated the optimal cutoff of VSIR using the R package (survminer). Student’s t-test and Kruskal-Walli’s test were performed to compare VSIR expression in the tumor and normal samples. In addition, the log-rank test was used to explore the survival differences between VSIR-high and VSIR-low groups regarding OS and DSS. All tests were two-sided, and *p* < 0.05 was statistically significant.

## Results

### VSIR Expression and Mutation Characteristics in Normal and Cancer Samples

**FIGURE 1:**
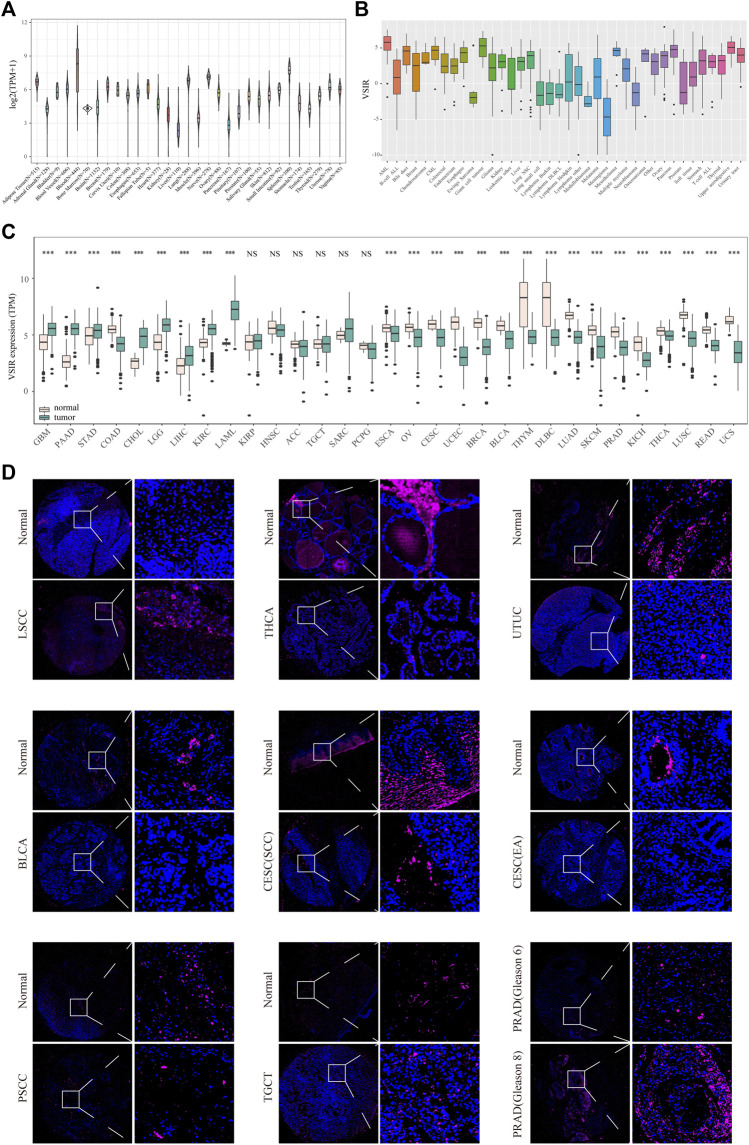
Expression profiles of VSIR in the normal and tumor tissues. VSIR levels in the 31 human tissues **(A)** and tumor cell lines **(B)**. VSIR levels in the tumor and normal samples were analyzed by the R language **(C)**. VSIR expression was revealed by immunofluorescence staining **(D)**. ***p < 0.001, NS: no significant differences.

First, to comprehensively describe the expression profile of VSIR in normal and cancer specimens, we download the RNA-seq data from three public databases. It was found that VSIR is expressed in 31 normal tissues in the GTEx database, among which the top five tissues with the highest VSIR content are blood, spleen, nerve, lung, and adipose tissue (Figure 1A). Results from the CCLE database showed the differential expression profile of VSIR in 38 tumor cell lines, among which the top three cell lines enriched by VSIR are AML, giant cell tumor, and upper aerodigestive (Figure 1B). We also compared the VSIR levels in the tumor samples and counterparts based on the GTEx and TCGA databases (Figure 1C). VSIR is significantly upregulated in the tumor than normal samples in GBM, STAD, CHOL, LIHC, pancreatic adenocarcinoma (PAAD), brain lower-grade glioma (LGG), kidney renal clear cell carcinoma (KIRC), and acute myeloid leukemia (LAML) (P<0.001). On the contrary, VSIR is significantly decreased (P<0.001) in the tumor than normal samples in OV, THYM, SKCM, PRAD, BRCA, esophageal carcinoma (ESCA), cervical squamous cell carcinoma (CESC), uterine corpus endometrial carcinoma (UCEC), bladder urothelial carcinoma (BLCA), lymphoid neoplasm diffuse large B-cell lymphoma (DLBC), lung adenocarcinoma (LUAD), kidney chromophobe (KICH), thyroid carcinoma (THCA), lung squamous cell carcinoma (LUSC), rectum adenocarcinoma (READ), and uterine carcinosarcoma (UCS) (P<0.001). These results were also verified through immunofluorescence staining (Figure 1D). In addition, we found that VSIR levels in the normal samples were higher than in cancer samples in upper tract urothelial cancer (UTUC) and Penile squamous cell carcinoma (PSCC), while laryngeal squamous cell carcinoma (LSCC) samples had higher VSIR expression than normal samples. Interestingly, PRAD samples with higher Gleason scores 8) have more VSIR than lower Gleason scores (6). Together with previous studies, these results help to illustrate the variable role of VSIR in different cancer types.

Meanwhile, we analyzed the mutation profiles of VSIR in pan-cancer using the cBioportal and GSCA datasets (Supplementary Figure S1). The results indicated that the top two cancers with the highest mutation levels are SKCM and STAD, with a mutation frequency above 4% (Supplementary Figures S1A, B). A total of 90 mutation sites (including seventy-five missenses, nine truncating, three splices, two fusions, and one inflame) were detected between amino acids 0 and 311 (Supplementary Figure S1C). We also summarized the survival profile between gene set copy number variation (CNV) groups in pan-cancer (Supplementary Figure S1D; Supplementary Table S1). There is a significant correlation between the survival time of LGG patients and gene set CNV (P<0.0001). The survival difference between gene set mutant (deleterious) and broad type in pan-cancer was also revealed in Supplementary Figure S1E; Supplementary Table S2.

### Prognostic Value of VSIR in Pan-Cancer

**FIGURE 2:**
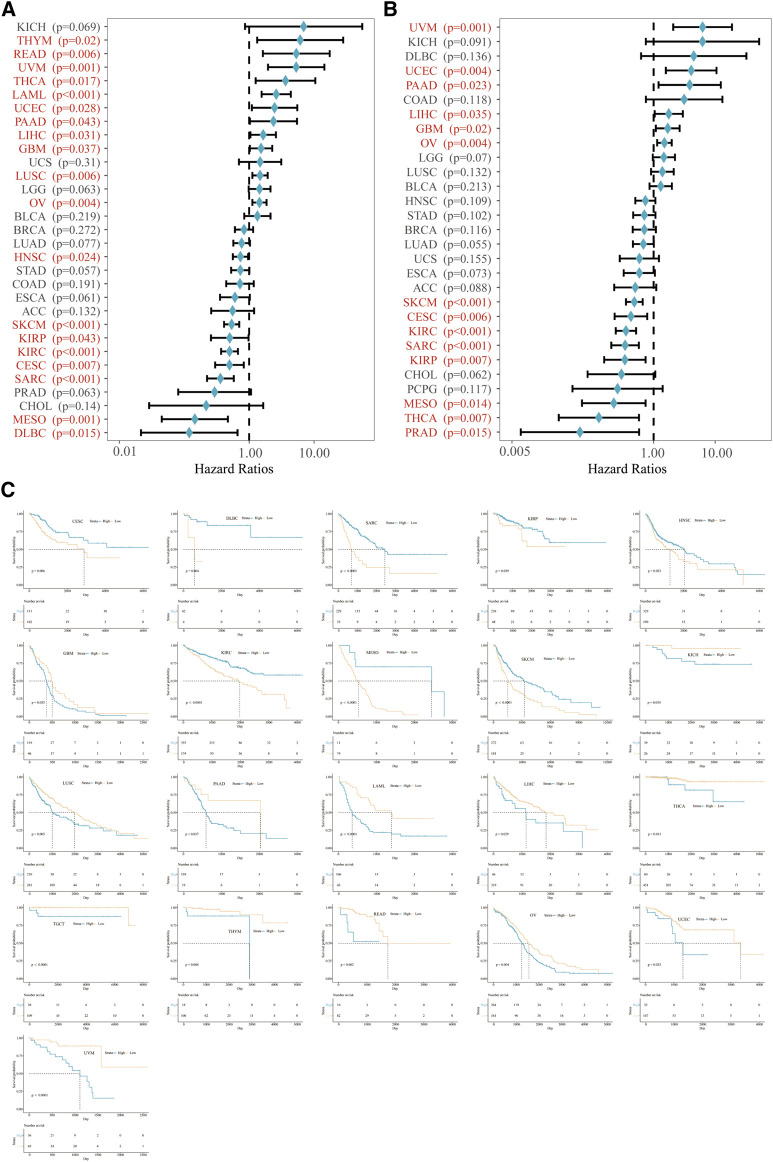
Prognostic value of VSIR in pan-cancer. Survival analysis of VSIR on OS **(A)** and DSS **(B)** in pan-cancer described by the forest plot. K. M. analysis of VSIR on OS **(C)**.

Next, we clarified the predictive value of VSIR in pan-cancer based on the KM analysis. VSIR was closely related to OS (Figure 2A) and DSS (Figure 2B) of multiple cancers. High VSIR levels are associated with longer OS than low VSIR levels in CESC, DLBC, SARC, kidney renal papillary cell carcinoma (KIRP), HNSC, KIRC, mesothelioma (MESO), and SKCM (Figure 2C; P<0.05). High VSIR expression, however, is related to shorter OS than low VSIR levels in GBM, KICH, LUSC, PAAD, LAML, LIHC, THCA, testicular germ cell tumor (TGCT), THYM, READ, OV, uveal melanoma (UVM), and UCEC (Figure 2C; P<0.05). In addition, we also found that elevated VSIR is associated with longer DSS in CHOL, KIRC, KIRP, MESO, PRAD, SARC, SKCM, and THCA (Supplementary Figure S2; P<0.05). On the contrary, elevated VSIR is related to shorter DSS in GBM, LIHC, OV, PAAD, TGCT, UCEC, and UVM (Supplementary Figure S2; P<0.05).

### Immune Characteristics of VSIR in Pan-Cancer TME

**FIGURE 3:**
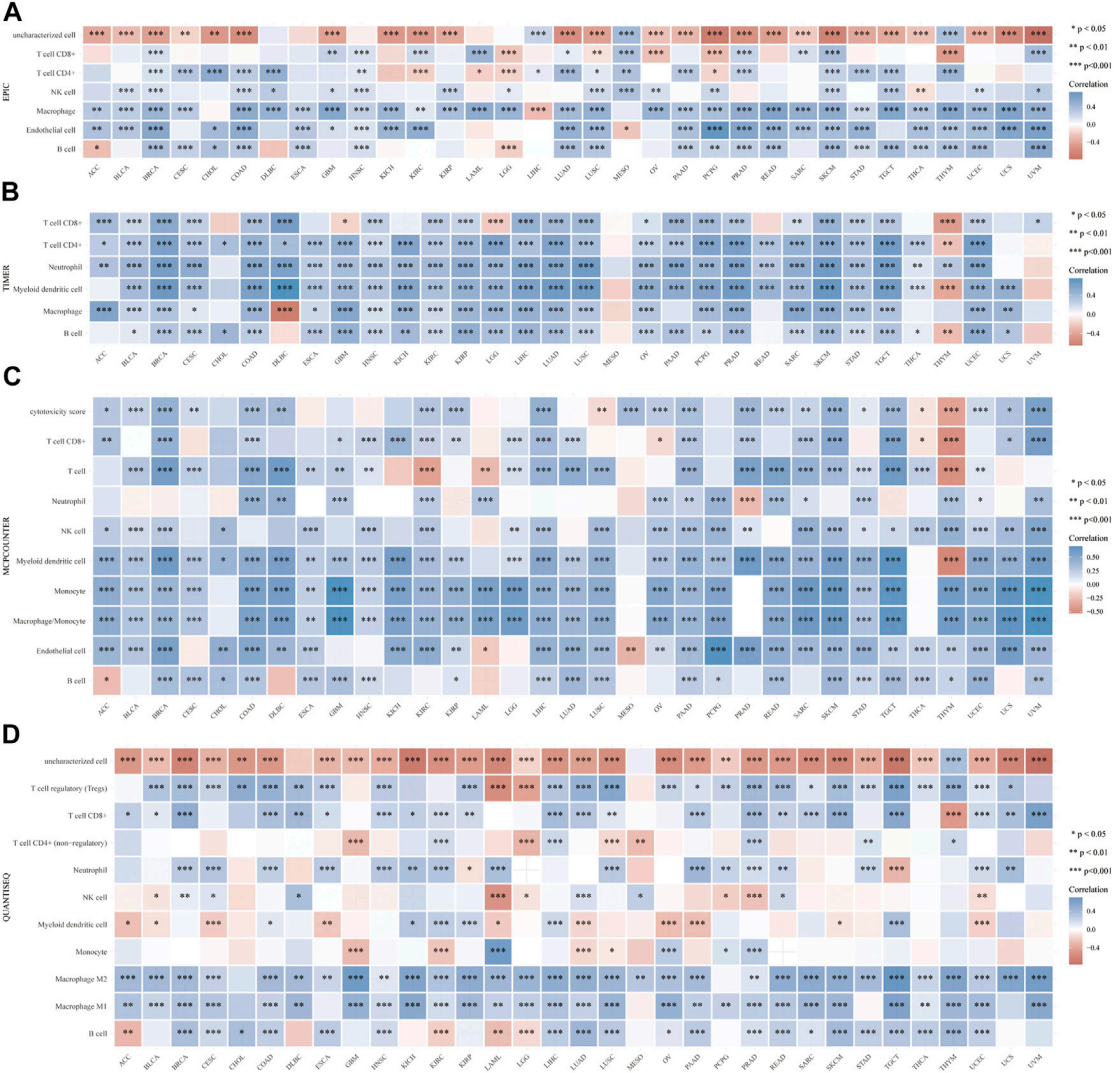
Correlation between VSIR and infiltrated inflammatory cells analyzed by the immunedeconv algorithm. Immune cell infiltration analyzed by the EPIC **(A)**, TIMER **(B)**, MCP-counter **(C)**, quanTIseq **(D)** algorithms. *p < 0.05, **p < 0.01, ***p < 0.001.

Then, we explored the immune features of VSIR in the tumor microenvironment among these cancers. We found that VSIR levels are positively related to immune score (Supplementary Figure S3), estimate score (Supplementary Figure S4), and stromal score (Supplementary Figure S5) in almost all cancers. Especially BLCA, BRCA, and CESC are the top three cancers most related to immune and estimate scores (P<0.001); BLCA, BRCA, and COAD are the top three cancers most related to stromal score (P<0.001). Furthermore, it was found that VSIR expression is most positively correlated with immune infiltrates (B cell, CD4^+^, and CD8^+^ T cells, DCs, macrophages, and neutrophils) in ACC, BRCA, and CHOL (Supplementary Figure S6A; P<0.05). In addition, we also studied the relationship between VSIR expression and other infiltrated inflammatory cells in pan-cancer using six latest algorithms, including EPIC (Figure 3A), TIMER (Figure 3B), MCP-counter (Figure 3C), quanTIseq (Figure 3D), CIBERSORT (Supplementary Figure S6B), and xCell (Supplementary Figure S6C). VSIR expression was positively related to various infiltrated inflammatory cells in most cancer types. Increased VSIR kept a significant correlation with B cell, T cell, macrophages, neutrophils, NK Cells and monocytes in BLCA, BRCA, COAD, CESC, LGG, GBM, HNSC, KICH, KIRC, LIHC, LUAD, PAAD, PCPG, READ, SKCM, STAD, TGCT, and UCEC (P<0.05).

**FIGURE 4:**
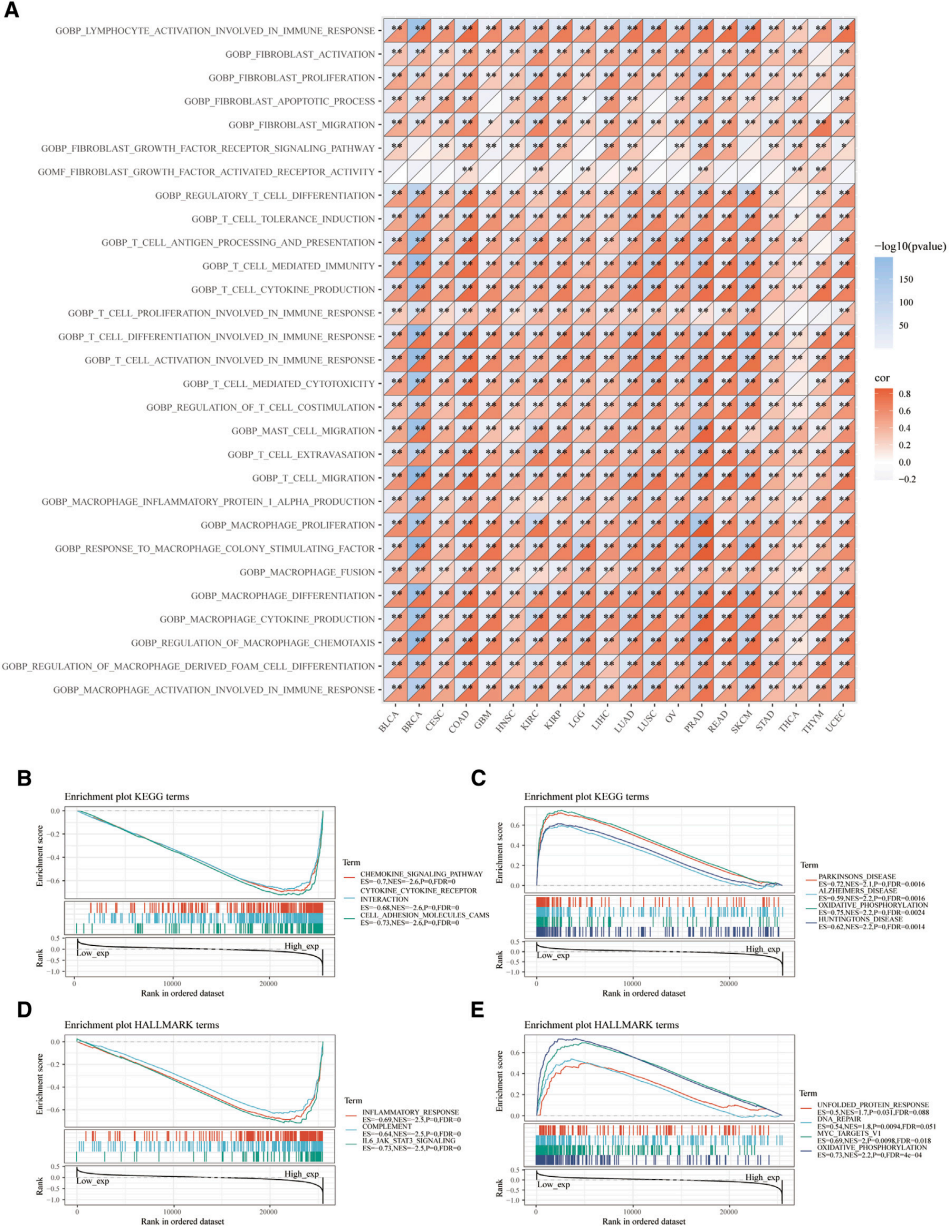
Functional pathways analysis of VSIR in pan-cancer. Enrichment pathways analysis of VSIR from the GSVA algorithm **(A)**. Top three negative **(B)** and top four positive **(C)** enrichment pathways of VSIR from the KEGG terms. Top three negative **(D)** and top four positive **(E)** enrichment pathways of VSIR from the HALLMARK terms.

Moreover, we demonstrated signaling pathways related to VSIR expression based on GOBP terms. A series of immune-related signaling pathways closely related to VSIR were discovered in pan-cancer, such as activation of lymphocytes involved in immune response, infiltrated inflammatory cells (fibroblast, T cell, mast cell, macrophages, and foam cell) activation, proliferation, and migration (Figure 4A; P<0.05). Based on the KEGG gene set, the top three negative enriched pathways were chemokine signaling, cytokine-cytokine receptor interaction, and cell adhesion molecules CAMs (Figure 4B; P<0.001); the top four positive enriched pathways were Parkinson’s disease, Alzheimer’s disease, oxidative phosphorylation, and Huntington’s disease (Figure 4C; P<0.001). This result indicated that VSIR might play a crucial role in CNS diseases through various immune-related pathways. Based on the HALLMARK gene set, the top three negative enriched pathways were inflammatory response, complement, and IL-6/JAK/STAT3 signaling (Figure 4D; P<0.001); the top four positive enriched pathways were unfolded protein response, DNA repair, MYC targets V1, and oxidative phosphorylation (Figure 4E; P<0.001).

Meanwhile, we calculated the relationship between VSIR expression and other immune checkpoints using Sangerbox (Supplementary Figure S7A) and immunedeconv (Supplementary Figure S7B) algorithms. Results indicated that VSIR levels significantly positively correlated with these immune checkpoints in many cancers, especially in BRCA, COAD, LIHC, SKCM, PRAD, TGCT, and UVM (P<0.05). SIGLEC15, IDO1, CD274, HAVCR2, PDCD1, CTLA-4, LAG3, and PDCD1LG2 are transcripts related to immune checkpoints. We found that VSIR was closely correlated with these markers in most cancers, especially in BRCA, COAD, LUAD, LUSC, PAAD, PRAD, and SKCM (P<0.05). VSIR levels in COAD are positively correlated with microsatellite instability (MSI) (P<0.0001), while negatively related to MSI in HNSC, LUSC, MESO, PCPG, READ, PRAD, SKCM, STAD, and TGCT (Supplementary Figures S8A, C; P<0.05). VSIR levels are positively correlated with tumor mutation burden (TMB) in COAD, SARC, UVM, and THYM(P<0.01), while negatively related to TMB in DLBC, GBM, LAML, LIHC, LUAD, LUSC, PRAD, SARC, SKCM, STAD, and THCA (Supplementary Figures S8B, D; P<0.05). VSIR levels are closely related to expressions of mismatch repair system genes (MMRs) in many cancers, such as BRCA, KIRC, LGG, and LIHC (Supplementary Figure S8E; P<0.01). VSIR was associated with neoantigen numbers in BRCA, STAD, THCA, BLCA, and PRAD (Supplementary Figure S9; P<0.05).

**FIGURE 5:**
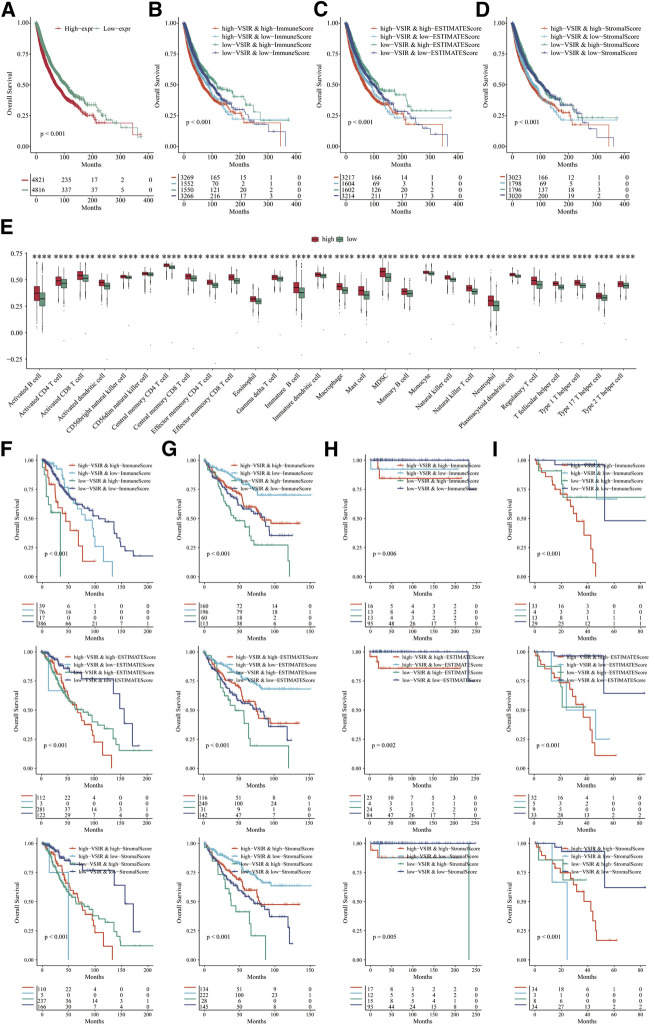
Role of VSIR in pan-cancer regarding VSIR expression, prognostic role, and association with tumor immunity. Survival analysis of VSIR in pan-cancer based on VSIR levels **(A)**, immune score **(B)**, estimate score **(C)**, stromal score **(D)**. Immune infiltrates in pan-cancer based on VSIR levels **(E)**. Survival analysis of VSIR regarding expression and score in LGG **(F)**, KIRC **(G)**, TGCT **(H)** and UVM **(I)**. ****p < 0.0001.

Furthermore, to comprehensively clarify the predictive value of VSIR in pan-cancer, we merged all the cancer datasets we used in this study and divided them into two groups regarding VSIR expression, prognostic role, and association with tumor immunity (Figure 5; Supplementary Figures S10). Results showed that low expression of VSIR correlates with longer OS and predicts a better prognosis in pan-cancer (Figure 5A; P<0.001). In the group with high VSIR expression, high immune score (Figure 5B; P<0.001), estimate score (Figure 5C; P<0.001) and stromal score (Figure 5D; P<0.001) indicate poor outcome in pan-cancer. We also explored the immune infiltrates in the TME based on VSIR levels in pan-cancer. A large number of immune cell types were found to upregulate in the VSIR high expression group, such as activated B cell, T cells, eosinophil, mast cell, macrophage, monocyte, NK cell and neutrophil (Figure 5E; P<0.0001). High scores are also associated with unfavorable prognosis in LGG (Figure 5F; P<0.01), favorable prognosis in KIRC (Figure 5G; P<0.05), unfavorable prognosis in TGCT (Figure 5H; P<0.05), unfavorable prognosis in UVM (Figure 5I; P<0.05). These results revealed that the extent to which VSIR correlates with immune cell infiltration can be used to explain prognostic outcomes.

### Co-Expression of VSIR With Tumor and Inflammatory Cells

**FIGURE 6:**
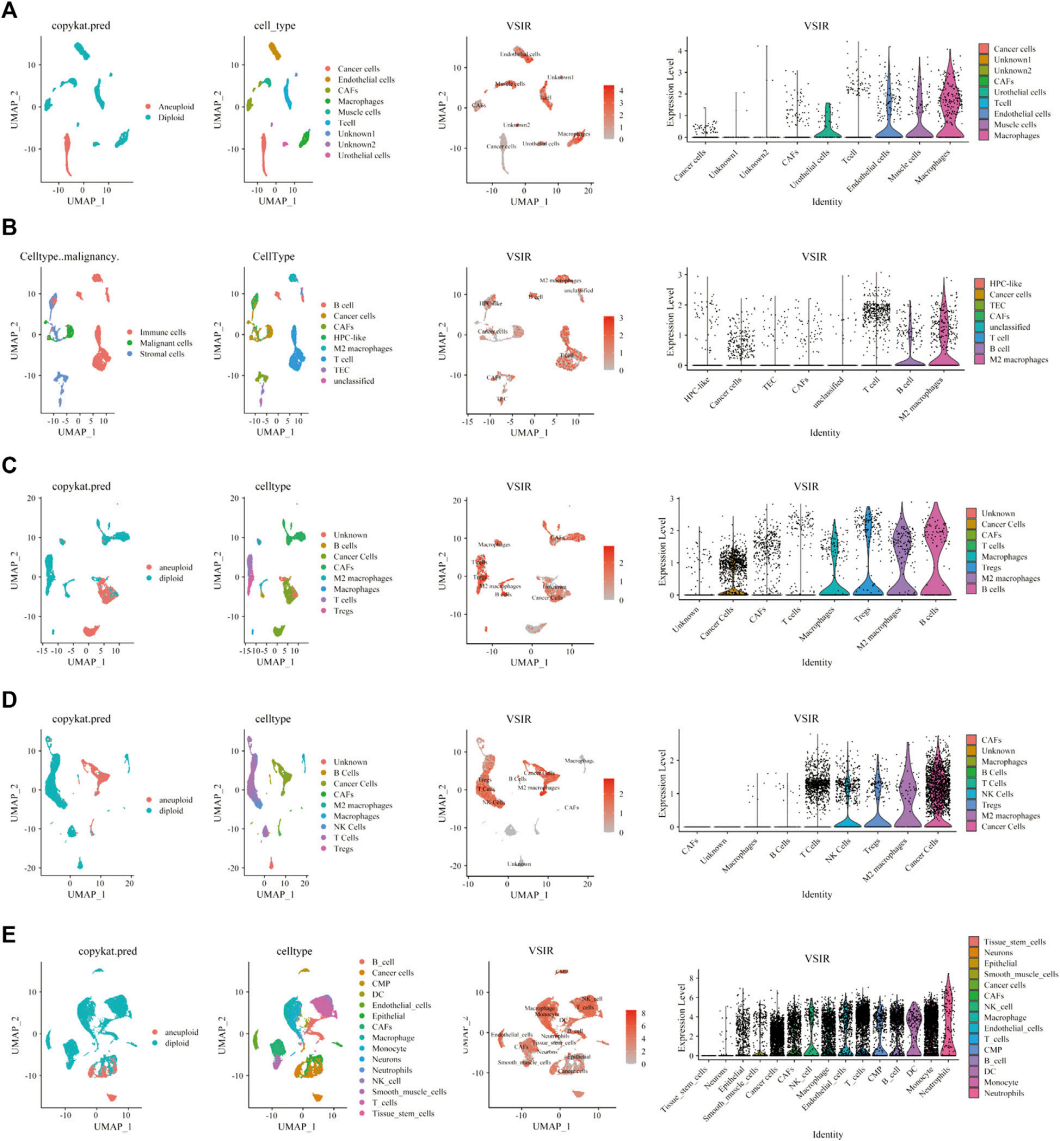
Single-cell sequencing analysis of VSIR in the TME. The relationship between VSIR levels and cancer cells and stromal cells in BLCA **(A)**, CHOL (B), HNSC **(C)**, KIRC **(D)**, LUAD **(E)**.

**FIGURE 7:**
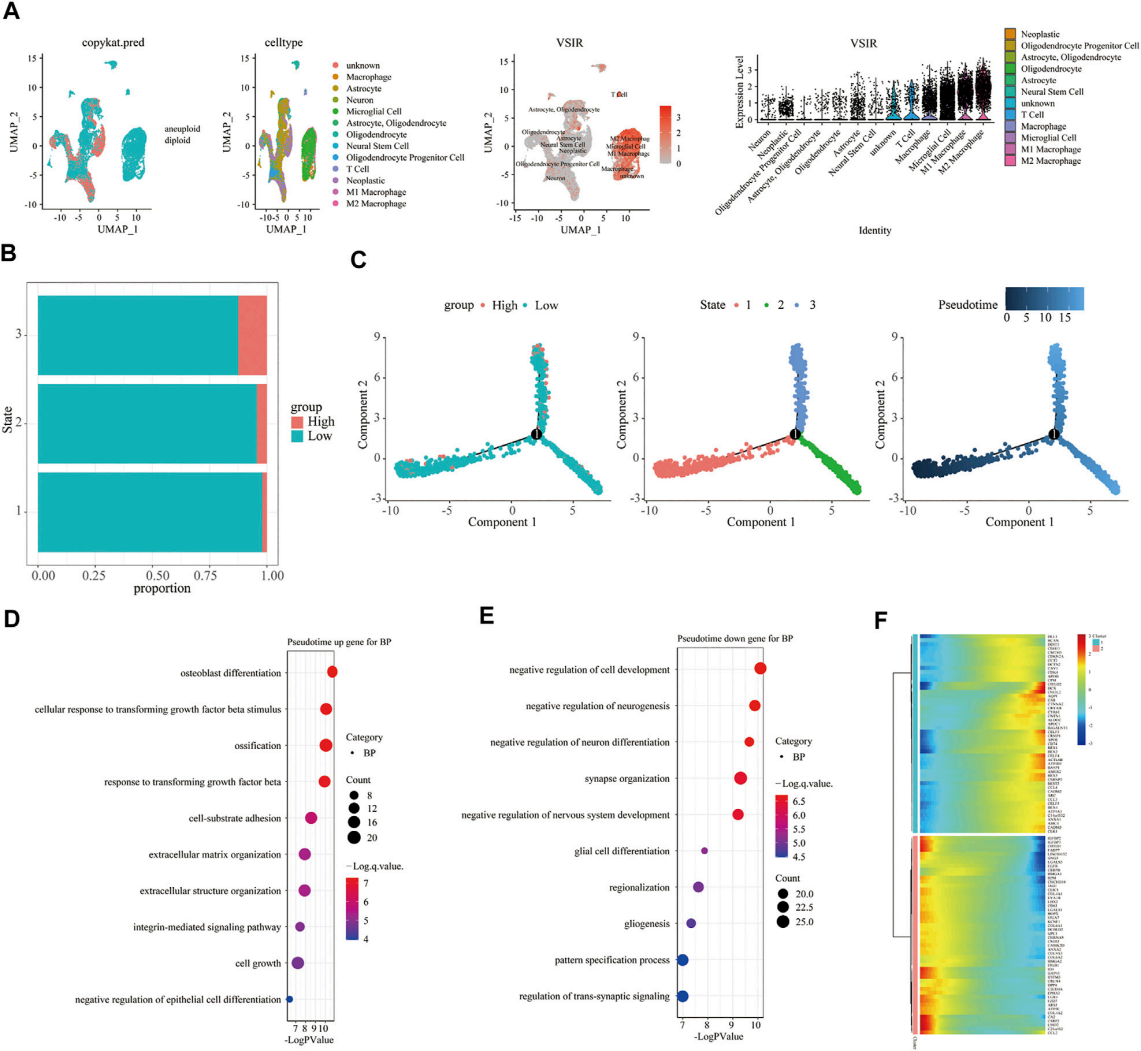
Immune characteristics of VSIR in the TME in GBM. Immune features of VSIR in the TME were explored by the single-cell sequencing analysis **(A)** and pseudotime trajectory analysis **(B, C)**. GO enrichment analysis revealed pathways based on the upregulated genes **(D)** and downregulated genes **(E)**. In addition, the top 100 downregulated and upregulated genes increase pseudotime **(F)**.

To fully clarify the role of VSIR in the TME in pan-cancer, we used single-cell sequencing analysis to observe the co-expression of VSIR with tumor and inflammatory cells in BLCA (Figure 6A), CHOL (Figure 6B), HNSC (Figure 6C), KIRC (Figure 6D), LUAD (Figure 6E), GBM (Figure 7A), BRCA (Supplementary Figure S11A), COAD (Supplementary Figure S11B), LIHC (Supplementary Figure S11C), and STAD (Supplementary Figure S11D). VSIR was found to co-expression with cancer cells and various inflammatory cells, such as T cells, B cells, regulatory T cells (Tregs), macrophages, M2 macrophages, cancer-associated fibroblasts (CAFs), monocytes, microglial cells, and astrocytes. Our previous enrichment pathway analysis indicated that VSIR might play an important role in CNS diseases, so we focused on the part of VSIR in BGM (Figure 7). We performed and defined three cell states using pseudotime trajectory analysis, in which VSIR expression increased as pseudotime increased (Figure 7B). VSIR was most expressed in state three compared with states 1 and 2 (Figure 7C). G.O. enrichment analysis revealed that as pseudotime increased, the top 10 signaling pathways enriched by upregulated genes are shown in Figure 7D. The top 10 signaling pathways enriched by downregulated genes are shown in Figure 7E. The top 100 downregulated and upregulated genes with the increase in pseudotime are shown in Figure 7F.

**FIGURE 8:**
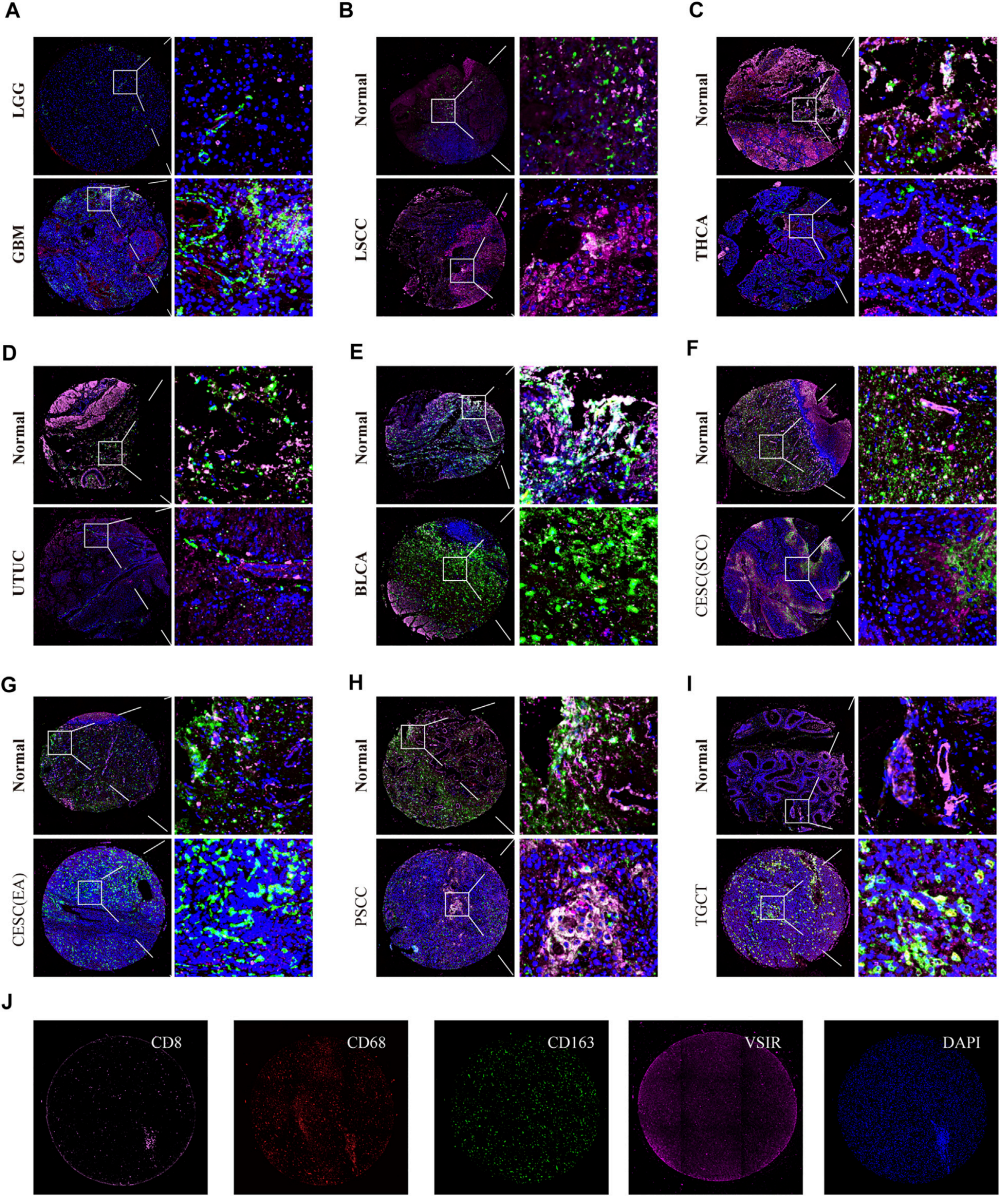
Triple immunofluorescence analyzes the co-expression of VSIR on T cells, macrophage, and M2 macrophage. The co-expression of VSIR with CD8^+^, CD68^+^, and CD163 + cells in LGG and GBM **(A)**, LSCC **(B)**, THCA **(C)**, UTUC **(D)**, BLCA **(E)**, CESC **(F, G)**, PSCC **(H)**, TGCT **(I)**. Immunofluorescence staining of CD8, CD68, CD163, VSIR and DAPI **(J)**.

We also observed the co-expression of VSIR with T cells (CD8), macrophages (CD68), and M2 macrophages (CD163) in GBM (Figure 8A), LSCC (Figure 8B), THCA (Figure 8C), UTUC (Figure 8D), BLCA (Figure 8E), CESC (Figures 8F,G), PSCC (Figure 8H), and TGCT (Figure 8I) using multiplex immunofluorescence staining (Figure 8J). VSIR was found to co-expression with CD163^+^ cells in these cancers, including GBM, LSCC, BLCA, CESC, PSCC, and TGCT. In addition, VSIR was found to co-expression with CD8^+^ in LSCC and PSCC.

### Immunotherapy Value of VSIR in Pan-Cancer

**FIGURE 9:**
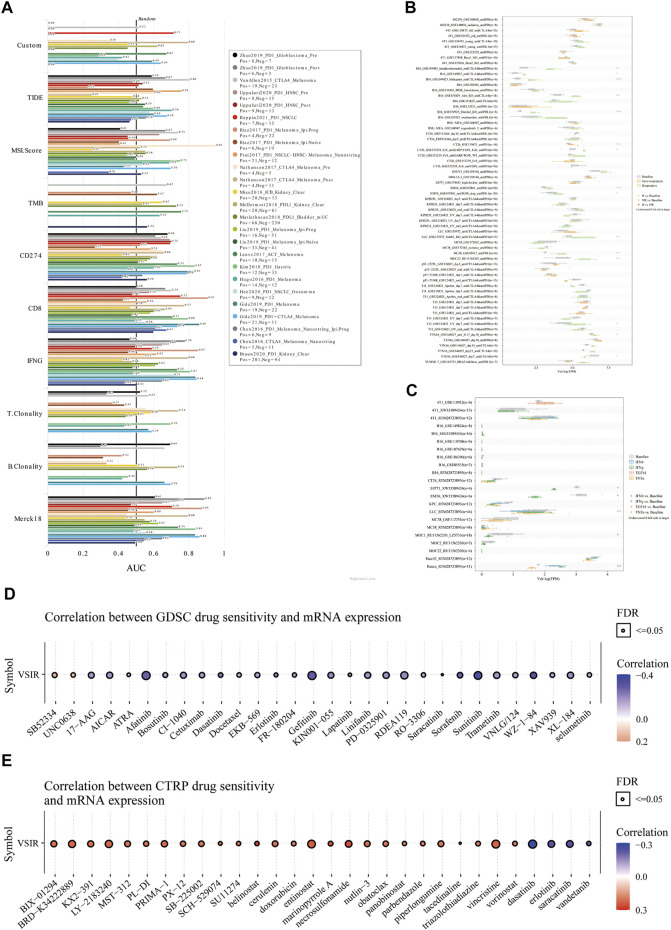
Immunotherapeutic value of VAIR in pan-cancer. The predictive value of VSIR in 25 immunotherapy cohorts **(A)**. Immunotherapy response of VSIR in murine immunotherapy cohorts based on *in vivo* studies **(B)** and *in vitro* studies **(C)**. Correlation between VSIR expression and drug sensitivity from the GDSC **(D)** and CTRP **(E)** datasets.

Finally, we predicted the immunotherapy value of VSIR in pan-cancer based on the public databases. Biomarker relevance of VSIR was calculated by comparing it with standardized biomarkers based on their predictive power of response outcomes and OS of human immunotherapy cohorts. VSIR alone had an AUC of more than 0.5 in seven of 25 immunotherapy cohorts (Figure 9A). VSIR has the similar predictive value with B. Clonality (AUC >0.5 in seven immunotherapy cohorts). However, the predictive value of VSIR was lower than the MSI score, CD274, TIDE, IFNG, and CD8, which respectively gave AUC values above 0.5 in 13, 21, 18, 17, 18 immunotherapy cohorts. Interestingly, VSIR was found to significantly predict immunotherapy response in 15 murine immunotherapy cohorts based on *in vivo* studies (Figure 9B) and five murine cytokine treatment cohorts based on *in vitro* studies (Figure 9C). We then predicted sensitive small molecules and drugs based on VSIR levels through the public databases. The expression of each gene in the gene set was performed by Spearman correlation analysis with the small molecule/drug sensitivity (IC50). The positive correlation means that the gene is resistant to the drug and the negative correlation means that the gene is sensitive to the drug. Many sensitive drugs negatively correlated with VSIR expression were found based on the GDSC database. The top three drugs are sunitinib, WZ-1-84, and gefitinib (Figure 9D and Supplementary Table S3; *p* < 0.0001). Many resistant drugs positively correlated with VSIR expression were found based on the CTRP database. The top three drugs are belinostat, necrosulfonamide, and marinopyrrole A (Figure 9E and Supplementary Table S4; *p* < 0.0001).

## Discussion

In this paper, we discussed the expression profile and prognostic value of VSIR in pan-cancer. VSIR levels are higher in the tumor than normal samples in GBM, STAD, CHOL, LIHC, PAAD, LGG, KIRC, and LAML, while being lower in OV, THYM, SKCM, PRAD, BRCA, ESCA, CESC, UCEC, BLCA, DLBC, LUAD, KICH, THCA, LUSC, READ, UCS. Meanwhile, we found that VSIR has an excellent predictive value in these cancers, which will provide new directions for future research on the function of VSIR in tumor immunity in these cancers.

Tumor-infiltrating inflammatory cells play a vital role in the progress of tumor formation, growth, metastasis, and recurrence. For example, As an indispensable component for mediating immune homeostasis, Tregs play essential roles in tumor immunity by inhibiting the activation and differentiation of CD4^+^ helper T cells and CD8^+^ cytotoxic T cells (Newton et al., 2016). In the progress of tumor immune response, Tregs differentiated from ordinary T cells can secrete TGF-β, IL-10, and IL-35, which suppress antitumor immunity and promote the formation and progression of tumors (Li et al., 2020). Recently, attempts to target CAFs for benefit have become a promising way to improve cancer therapy. CAFs regulate cancer metastasis through a series of complex mechanisms, including rebuilding the ECM, inducing growth factors, influencing drug resistance and immunotherapy response, remodeling the interactions with cancer cells, and infiltrating inflammatory cells (Sahai et al., 2020). In this study, we clarified the correlation between VSIR expression and infiltrated immune cells in the TME among these cancers through public databases, single-cell sequencing analysis, and multiplex immunofluorescence staining. Tumor-associated macrophages (TAMs) regulate inflammation and initiate, facilitate, or inhibit cancer development by affecting other immune cells and producing various factors, including nitric oxide, VEGF, EGF, and TGF-β(Zhang et al., 2021b; Duan and Luo, 2021; Zhang N. et al., 2021). VSIR plays a key role in reprogram and restrain macrophage inflammatory differentiation by regulating factors that mediate macrophage tolerance and inflammation during immune response. Anti-VSIR antibody treatment was found to induce mediators involved in both M2 macrophages polarization (up-regulated IL-10, miR-221, A20 and IRG1) and suppress mediators of M1 macrophages polarization (down-regulated IRF5 and IRF8) (ElTanbouly et al., 2020). scRNA-seq technology analysis indicated that macrophages derived from VSIR^−/-^ mice and macrophages derived from WT mice exhibit heterogeneity in certain subtypes and have different functions. Especially, the expression levels of Hspb1, Cebpb, Gm8797, Bag3 and interferon-stimulated gene Ifit1 were upregulated in the macrophages from VSIR^−/-^ mice skin lesions compared to WT controls (Qie et al., 2020). Increasing studies have found that according to different subtypes (anti-tumorigenic neutrophils and pro-tumorigenic neutrophils), tumor-associated neutrophils (TANs) play diametrically opposite effects in tumor immunity (Galli et al., 2020). It was found that VSIR maintains a close relationship with these inflammatory cells in most cancers, especially with T cells, CAFs, macrophages, M2 macrophages, Tregs, neutrophils, monocytes, microglial cells, and astrocytes. VSIR was found to engage in several immune-related signaling pathways, such as regulating the activation, proliferation, and migration of fibroblast, T cell, mast cell, macrophages, and foam cell. In addition, the co-expression of VSIR on M2 macrophages and T cells was also detected in our tumor chip.

The B7 protein is expressed on activated antigen-presenting cells and can bind to classical immune checkpoints on T cells, such as CD28, PD-L1, and CTLA-4 (Chaudhri et al., 2018; Du et al., 2018). Structural analysis revealed similar functional domains between VSIR and B7 family members, containing a conserved IGV-like fold. Phylogeny and protein sequence analysis show that VSIR is also partially homologous to PD-1 and CTLA-4 (Nowak et al., 2017). Thus, the VSIR signaling pathway has increasingly become a promising immunotherapy target in anti-cancer treatment in recent years. Although the VSIR antagonist’s monotherapy did not show any effect based on preclinical studies, the combination of VSIR and CTLA-4 or PD-1 significantly reduced the number of tumor-recruiting T cells, thereby inducing antitumor responses against squamous cell carcinoma (Kondo et al., 2016). In addition, studies have found that patients who show resistance to PD-1/CTLA-4-targeting therapies may benefit from VSIR blockade (Kong et al., 2021). Moreover, CA-170, an orally administered dual inhibitor of VISTA and PD-L1, has shown clinical efficacy in phase I and II clinical trials in several advanced solid tumor types (Sasikumar et al., 2021). This paper comprehensively explored the relationship between VSIR and other immune checkpoints. We found that VSIR exhibited a close positive correlation with these immune checkpoints in most cancers, especially with LAIR1, HAVCR2, SIGLEC15, IDO1, CD274, HAVCR2, PDCD1, CTLA-4, LAG3, and PDCD1LG2. In addition, we discovered that VSIR expression is positively associated with the immune score, estimate score, and stromal score in these cancers. In addition, to fully clarify the immune aspects of VSIR in pan-cancer, we merged all the samples we used in this study and divided them into two groups based on VSIR levels and scores. We detected increased expression of a large number of infiltrating immune cells in the VSIR high expression group, including activated B cells, T cells, eosinophils, mast cells, macrophages, monocytes, NK cells and neutrophils. In the high VSIR expression group, patients with high immune, estimate and stromal score had poorer outcome in pan-cancer, especially in LGG, TGCT, and UVM. These results re-emphasize that irrespective of the difference in levels of its expression, VSIR is significantly associated with tumor immunity across different cancers. MMRs are a group of particular genes that identify genetic error pairs and initiate repair (Tamura et al., 2019). We found that VSIR levels are closely related to mutations of mismatch repair system genes (MMRs) in several cancers, such as BRCA, KIRC, LGG, and LIHC. Tumor neoantigens, including antigens produced by cancer cells and antigens produced by mutant proteins, have been considered a new approach to cancer immunotherapy (Zhang Z. et al., 2021). VSIR was associated with neoantigen numbers in BRCA, STAD, THCA, BLCA, and PRAD. Taking these data together, we speculate that VSIR plays a pivotal role in tumor immunity by affecting the functions of many inflammatory cells and can be used as a novel immunotherapy target in the process of tumor treatment in the future.

The past few decades have witnessed exciting breakthroughs that help us understand tumor immunity’s specific mechanisms and pathways (Ventola, 2017). However, even with these advances, cancer immunotherapy still has limitations. For example, obstacles such as the inability to predict immunotherapy efficacy and patient response, the lack of sensitive drugs and development of treatment resistance, and the difficulty in finding the optimal biomarkers are still the main factors hindering the benefit of cancer patients. Therefore, in the last of this paper, we identified the therapeutic value of VSIR in 25 immunotherapy cohorts through the public databases. We found that VSIR alone had predictive value in seven of 25 immunotherapy cohorts. Furthermore, in several immunotherapy cohorts, VSIR has the similar predictive value with B. Clonality. However, the predictive value of VSIR was lower than the MSI score, CD274, TIDE, IFNG, and CD8 in more than 10 immunotherapy cohorts. Of note, we found VSIR can predict immunotherapy response in 15 murine immunotherapy cohorts based on *in vivo* studies and five murine immunotherapy cohorts based on *in vitro* studies. Furthermore, a series of sensitive small molecules and drugs were found based on the GDSC and CTRP database, further increasing the benefits of targeting the highly expressed VSIR in the TME in tumor immunotherapy. The top five sensitive drugs were sunitinib, WZ-1-84, gefitinib, afatinib, and sorafenib based on the VSIR expression from the GDSC database. The top three drugs positively correlated with VSIR expression were LY-2183240, vincristine, and entinostat, which means VSIR is resistant to these drugs. For example, afatinib, gefitinib, and sunitinib are EGFR inhibitors, which have been confirmed by many clinical trials, which can significantly inhibit the growth, metastasis and angiogenesis of various cancers, and increase the apoptosis of tumor cells. Especially for patients with the NSCL, HNSC, MESO, KIRC, leukemia, and KIRC, these drugs can significantly benefit them and prolong the time of tumor recurrence (George et al., 2009; Bang et al., 2011; Rizzo and Porta, 2017; Deeks and Keating, 2018). Excitingly, our results indicated that elevated VSIR levels are associated with better outcome than low VSIR levels in CESC, DLBC, SARC, KIRP, HNSC, KIRC, MESO, and SKCM. These data provide a new perspective for us to expand the therapeutic range of these drugs and develop new targeted drugs for those tumors whose prognosis is correlated with VSIR expression.

It should be noted that in cancer types with high infiltration of M2 macrophages and high expression of VSIR, patients with the corresponding cancers were associated with decreased survival, which might also partly explain the observation that VSIR behaves similarly in some cancers. Likewise, in cancer types with weakened activation of immune response and high expression of VSIR, patients with the corresponding cancers were almost uniformly associated with decreased survival. Notably, in cancer types with the negative correlation between VSIR and TMB, patients with the corresponding cancers were also associated with decreased survival. Taken together, VSIR was likely to induce an immune suppressive microenvironment in some cancers, which could lead to the tumor progression and decreased survival of patients.

## Data Availability

The datasets presented in this study can be found in online repositories. The names of the repository/repositories and accession number(s) can be found in the article/Supplementary Material.

## References

[B1] Arnold M. Rutherford M. J. Bardot A. Ferlay J. Andersson T. M.-L. Myklebust T. Å. (2019). Progress in Cancer Survival, Mortality, and Incidence in Seven High-Income Countries 1995-2014 (ICBP SURVMARK-2): a Population-Based Study. Lancet Oncol. 20 (11), 1493–1505. 10.1016/S1470-2045(19)30456-5 31521509 PMC6838671

[B2] Bach A. C. Lo K. S. Pathirana T. Glasziou P. P. Barratt A. L. Jones M. A. (2020). Is the Risk of Cancer in Australia Overstated? the Importance of Competing Mortality for Estimating Lifetime Risk. Med. J. Aust. 212 (1), 17–22. 10.5694/mja2.50376 31691294

[B3] Baghban R. Roshangar L. Jahanban-Esfahlan R. Seidi K. Ebrahimi-Kalan A. Jaymand M. (2020). Tumor Microenvironment Complexity and Therapeutic Implications at a Glance. Cell Commun Signal 18 (1), 59. 10.1186/s12964-020-0530-4 32264958 PMC7140346

[B4] Bang Y.-J. Kang Y.-K. Kang W. K. Boku N. Chung H. C. Chen J.-S. (2011). Phase II Study of Sunitinib as Second-Line Treatment for Advanced Gastric Cancer. Invest. New Drugs 29 (6), 1449–1458. 10.1007/s10637-010-9438-y 20461441 PMC3171673

[B5] Butler A. Hoffman P. Smibert P. Papalexi E. Satija R. (2018). Integrating Single-Cell Transcriptomic Data across Different Conditions, Technologies, and Species. Nat. Biotechnol. 36 (5), 411–420. 10.1038/nbt.4096 29608179 PMC6700744

[B6] Chaudhri A. Xiao Y. Klee A. N. Wang X. Zhu B. Freeman G. J. (2018). PD-L1 Binds to B7-1 Only in Cis on the Same Cell Surface. Cancer Immunol. Res. 6 (8), 921–929. 10.1158/2326-6066.CIR-17-0316 29871885 PMC7394266

[B7] Davis C. Naci H. Gurpinar E. Poplavska E. Pinto A. Aggarwal A. (2017). Availability of Evidence of Benefits on Overall Survival and Quality of Life of Cancer Drugs Approved by European Medicines Agency: Retrospective Cohort Study of Drug Approvals 2009-13. BMJ 359, j4530. 10.1136/bmj.j4530 28978555 PMC5627352

[B8] Deeks E. D. Keating G. M. (2018). Afatinib in Advanced NSCLC: a Profile of its Use. Drugs Ther. Perspect. 34 (3), 89–98. 10.1007/s40267-018-0482-6 29540977 PMC5840214

[B9] Du X. Tang F. Liu M. Su J. Zhang Y. Wu W. (2018). A Reappraisal of CTLA-4 Checkpoint Blockade in Cancer Immunotherapy. Cell Res 28 (4), 416–432. 10.1038/s41422-018-0011-0 29472691 PMC5939050

[B10] Duan Z. Luo Y. (2021). Targeting Macrophages in Cancer Immunotherapy. Sig Transduct Target. Ther. 6 (1), 127. 10.1038/s41392-021-00506-6 PMC7994399 33767177

[B11] Dyck L. Mills K. H. G. (2017). Immune Checkpoints and Their Inhibition in Cancer and Infectious Diseases. Eur. J. Immunol. 47 (5), 765–779. 10.1002/eji.201646875 28393361

[B12] ElTanbouly M. A. Croteau W. Noelle R. J. Lines J. L. (2019). VISTA: a Novel Immunotherapy Target for Normalizing Innate and Adaptive Immunity. Semin. Immunol. 42, 101308. 10.1016/j.smim.2019.101308 31604531 PMC7233310

[B13] ElTanbouly M. A. Schaafsma E. Smits N. C. Shah P. Cheng C. Burns C. (2020). VISTA Re-programs Macrophage Biology through the Combined Regulation of Tolerance and Anti-inflammatory Pathways. Front. Immunol. 11, 580187. 10.3389/fimmu.2020.580187 33178206 PMC7593571

[B14] Flies D. B. Wang S. Xu H. Chen L. (2011). Cutting Edge: A Monoclonal Antibody Specific for the Programmed Death-1 Homolog Prevents Graft-Versus-Host Disease in Mouse Models. J.I. 187 (4), 1537–1541. 10.4049/jimmunol.1100660 PMC3150865 21768399

[B15] Galli F. Aguilera J. V. Palermo B. Markovic S. N. Nisticò P. Signore A. (2020). Relevance of Immune Cell and Tumor Microenvironment Imaging in the new era of Immunotherapy. J. Exp. Clin. Cancer Res. 39 (1), 89. 10.1186/s13046-020-01586-y 32423420 PMC7236372

[B16] Gao J. Navai N. Alhalabi O. Siefker-Radtke A. Campbell M. T. Tidwell R. S. (2020). Neoadjuvant PD-L1 Plus CTLA-4 Blockade in Patients with Cisplatin-Ineligible Operable High-Risk Urothelial Carcinoma. Nat. Med. 26 (12), 1845–1851. 10.1038/s41591-020-1086-y 33046869 PMC9768836

[B17] George S. Merriam P. Maki R. G. Van den Abbeele A. D. Yap J. T. Akhurst T. (2009). Multicenter Phase II Trial of Sunitinib in the Treatment of Nongastrointestinal Stromal Tumor Sarcomas. Jco 27 (19), 3154–3160. 10.1200/JCO.2008.20.9890 PMC2716937 19451429

[B18] He X. Xu C. (2020). Immune Checkpoint Signaling and Cancer Immunotherapy. Cel Res 30 (8), 660–669. 10.1038/s41422-020-0343-4 PMC7395714 32467592

[B19] Hosseinkhani N. Derakhshani A. Shadbad M. A. Argentiero A. Racanelli V. Kazemi T. (2021). The Role of V-Domain Ig Suppressor of T Cell Activation (VISTA) in Cancer Therapy: Lessons Learned and the Road Ahead. Front. Immunol. 12, 676181. 10.3389/fimmu.2021.676181 34093577 PMC8172140

[B20] Jahanban-Esfahlan R. Seidi K. Monhemi H. Adli A. D. F. Minofar B. Zare P. (2017). RGD Delivery of Truncated Coagulase to Tumor Vasculature Affords Local Thrombotic Activity to Induce Infarction of Tumors in Mice. Sci. Rep. 7 (1), 8126. 10.1038/s41598-017-05326-9 28811469 PMC5557930

[B21] Kondo Y. Ohno T. Nishii N. Harada K. Yagita H. Azuma M. (2016). Differential Contribution of Three Immune Checkpoint (VISTA, CTLA-4, PD-1) Pathways to Antitumor Responses against Squamous Cell Carcinoma. Oral Oncol. 57, 54–60. 10.1016/j.oraloncology.2016.04.005 27208845

[B22] Kong X. Lu P. Liu C. Guo Y. Yang Y. Peng Y. (2021). A Combination of PD-1/PD-L1 I-nhibitors: The prospect of O-vercoming the W-eakness of T-umor I-mmunotherapy (Review). Mol. Med. Rep. 23 (5). 10.3892/mmr.2021.12001 PMC7985997 33760188

[B23] Kuklinski L. F. Yan S. Li Z. Fisher J. L. Cheng C. Noelle R. J. (2018). VISTA Expression on Tumor-Infiltrating Inflammatory Cells in Primary Cutaneous Melanoma Correlates with Poor Disease-specific Survival. Cancer Immunol. Immunother. 67 (7), 1113–1121. 10.1007/s00262-018-2169-1 29737375 PMC11028124

[B24] Lánczky A. Győrffy B. (2021). Web-Based Survival Analysis Tool Tailored for Medical Research (KMplot): Development and Implementation. Med. Internet Res. 23 (7), e27633. 10.2196/27633 PMC8367126 34309564

[B25] Le Mercier I. Chen W. Lines J. L. Day M. Li J. Sergent P. (2014). VISTA Regulates the Development of Protective Antitumor Immunity. Cancer Res. 74 (7), 1933–1944. 10.1158/0008-5472.CAN-13-1506 24691994 PMC4116689

[B26] Le Mercier I. Lines J. L. Noelle R. J. (2015). Beyond CTLA-4 and PD-1, the Generation Z of Negative Checkpoint Regulators. Front. Immunol. 6, 418. 10.3389/fimmu.2015.00418 26347741 PMC4544156

[B27] Li C. Jiang P. Wei S. Xu X. Wang J. (2020). Regulatory T Cells in Tumor Microenvironment: New Mechanisms, Potential Therapeutic Strategies and Future Prospects. Mol. Cancer 19 (1), 116. 10.1186/s12943-020-01234-1 32680511 PMC7367382

[B28] Li T. Fan J. Wang B. Traugh N. Chen Q. Liu J. S. (2017). TIMER: A Web Server for Comprehensive Analysis of Tumor-Infiltrating Immune Cells. Cancer Res. 77 (21), e108–e110. 10.1158/0008-5472.CAN-17-0307 29092952 PMC6042652

[B29] Lim A. R. Rathmell W. K. Rathmell J. C. (2020). The Tumor Microenvironment as a Metabolic Barrier to Effector T Cells and Immunotherapy. Elife 9. 10.7554/eLife.55185 PMC7200151 32367803

[B30] Lines J. L. Pantazi E. Mak J. Sempere L. F. Wang L. O'Connell S. (2014). VISTA Is an Immune Checkpoint Molecule for Human T Cells. Cancer Res. 74 (7), 1924–1932. 10.1158/0008-5472.CAN-13-1504 24691993 PMC3979527

[B31] Liu C.-J. Hu F.-F. Xia M.-X. Han L. Zhang Q. Guo A.-Y. (2018). GSCALite: a Web Server for Gene Set Cancer Analysis. Bioinformatics 34 (21), 3771–3772. 10.1093/bioinformatics/bty411 29790900

[B32] Mulati K. Hamanishi J. Matsumura N. Chamoto K. Mise N. Abiko K. (2019). VISTA Expressed in Tumour Cells Regulates T Cell Function. Br. J. Cancer 120 (1), 115–127. 10.1038/s41416-018-0313-5 30382166 PMC6325144

[B33] Muller S. Victoria Lai W. Adusumilli P. S. Desmeules P. Frosina D. Jungbluth A. (2020). V-domain Ig-Containing Suppressor of T-Cell Activation (VISTA), a Potentially Targetable Immune Checkpoint Molecule, Is Highly Expressed in Epithelioid Malignant Pleural Mesothelioma. Mod. Pathol. 33 (2), 303–311. 10.1038/s41379-019-0364-z 31537897 PMC8366498

[B34] Newton R. Priyadharshini B. Turka L. A. (2016). Immunometabolism of Regulatory T Cells. Nat. Immunol. 17 (6), 618–625. 10.1038/ni.3466 27196520 PMC5006394

[B35] Nowak E. C. Lines J. L. Varn F. S. Deng J. Sarde A. Mabaera R. (2017). Immunoregulatory Functions of VISTA. Immunol. Rev. 276 (1), 66–79. 10.1111/imr.12525 28258694 PMC5702497

[B36] Ping Q. Yan R. Cheng X. Wang W. Zhong Y. Hou Z. (2021). Correction: Cancer-Associated Fibroblasts: Overview, Progress, Challenges, and Directions. Cancer Gene Ther. 28 (9), 1074. 10.1038/s41417-021-00343-3 34183778

[B37] Qie C. Jiang J. Liu W. Hu X. Chen W. Xie X. (2020). Single-cell RNA-Seq Reveals the Transcriptional Landscape and Heterogeneity of Skin Macrophages in Vsir-/- Murine Psoriasis. Theranostics 10 (23), 10483–10497. 10.7150/thno.45614 32929361 PMC7482809

[B38] Qin S. Xu L. Yi M. Yu S. Wu K. Luo S. (2019). Novel Immune Checkpoint Targets: Moving beyond PD-1 and CTLA-4. Mol. Cancer 18 (1), 155. 10.1186/s12943-019-1091-2 31690319 PMC6833286

[B39] Rizzo M. Porta C. (2017). Sunitinib in the Treatment of Renal Cell Carcinoma: an Update on Recent Evidence. Ther. Adv. Urol. 9 (8), 195–207. 10.1177/1756287217713902 29662544 PMC5896861

[B40] Sahai E. Astsaturov I. Cukierman E. DeNardo D. G. Egeblad M. Evans R. M. (2020). A Framework for Advancing Our Understanding of Cancer-Associated Fibroblasts. Nat. Rev. Cancer 20 (3), 174–186. 10.1038/s41568-019-0238-1 31980749 PMC7046529

[B41] Sasikumar P. G. Sudarshan N. S. Adurthi S. Ramachandra R. K. Samiulla D. S. Lakshminarasimhan A. (2021). PD-1 Derived CA-170 Is an Oral Immune Checkpoint Inhibitor that Exhibits Preclinical Anti-tumor Efficacy. Commun. Biol. 4 (1), 699. 10.1038/s42003-021-02191-1 34103659 PMC8187357

[B42] Siegel R. L. Miller K. D. Fuchs H. E. Jemal A. (2021). Cancer Statistics, 2021. CA A. Cancer J. Clin. 71 (1), 7–33. 10.3322/caac.21654 33433946

[B43] Siegel R. L. Miller K. D. Jemal A. (2020). Cancer Statistics, 2020. CA A. Cancer J. Clin. 70 (1), 7–30. 10.3322/caac.21590 31912902

[B44] Tamura K. Kaneda M. Futagawa M. Takeshita M. Kim S. Nakama M. (2019). Genetic and Genomic Basis of the Mismatch Repair System Involved in Lynch Syndrome. Int. J. Clin. Oncol. 24 (9), 999–1011. 10.1007/s10147-019-01494-y 31273487

[B45] Ventola C. L. (2017). Cancer Immunotherapy, Part 3: Challenges and Future Trends. P T 42 (8), 514–521. 28781505 PMC5521300

[B46] Villarroel-Espindola F. Yu X. Datar I. Mani N. Sanmamed M. Velcheti V. (2018). Spatially Resolved and Quantitative Analysis of VISTA/PD-1H as a Novel Immunotherapy Target in Human Non-small Cell Lung Cancer. Clin. Cancer Res. 24 (7), 1562–1573. 10.1158/1078-0432.CCR-17-2542 29203588 PMC5884702

[B47] Vitale I. Manic G. Coussens L. M. Kroemer G. Galluzzi L. (2019). Macrophages and Metabolism in the Tumor Microenvironment. Cel Metab. 30 (1), 36–50. 10.1016/j.cmet.2019.06.001 31269428

[B48] Yang K. Wu Z. Zhang H. Zhang N. Wu W. Wang Z. (2022). Glioma Targeted Therapy: Insight into Future of Molecular Approaches. Mol. Cancer 21 (1), 39. 10.1186/s12943-022-01513-z 35135556 PMC8822752

[B49] Yoon K. W. Byun S. Kwon E. Hwang S.-Y. Chu K. Hiraki M. (2015). Control of Signaling-Mediated Clearance of Apoptotic Cells by the Tumor Suppressor P53. Science 349 (6247), 1261669. 10.1126/science.1261669 26228159 PMC5215039

[B50] Zhang H. Dai Z. Wu W. Wang Z. Zhang N. Zhang L. (2021a). Regulatory Mechanisms of Immune Checkpoints PD-L1 and CTLA-4 in Cancer. J. Exp. Clin. Cancer Res. 40 (1), 184. 10.1186/s13046-021-01987-7 34088360 PMC8178863

[B51] Zhang H. Luo Y.-B. Wu W. Zhang L. Wang Z. Dai Z. (2021b). The Molecular Feature of Macrophages in Tumor Immune Microenvironment of Glioma Patients. Comput. Struct. Biotechnol. J. 19, 4603–4618. 10.1016/j.csbj.2021.08.019 34471502 PMC8383063

[B52] Zhang H. Wang Z. Dai Z. Wu W. Cao H. Li S. (2021c). Novel Immune Infiltrating Cell Signature Based on Cell Pair Algorithm Is a Prognostic Marker in Cancer. Front. Immunol. 12, 694490. 10.3389/fimmu.2021.694490 34594324 PMC8476752

[B53] Zhang J.-c. Chen W.-d. Alvarez J. B. Jia K. Shi L. Wang Q. (2018). Cancer Immune Checkpoint Blockade Therapy and its Associated Autoimmune Cardiotoxicity. Acta Pharmacol. Sin 39 (11), 1693–1698. 10.1038/s41401-018-0062-2 29991709 PMC6289335

[B54] Zhang N. Zhang H. Wang Z. Dai Z. Zhang X. Cheng Q. (2021d). Immune Infiltrating Cells-Derived Risk Signature Based on Large-Scale Analysis Defines Immune Landscape and Predicts Immunotherapy Responses in Glioma Tumor Microenvironment. Front. Immunol. 12, 691811. 10.3389/fimmu.2021.691811 34489938 PMC8418124

[B55] Zhang Z. Lu M. Qin Y. Gao W. Tao L. Su W. (2021e). Neoantigen: A New Breakthrough in Tumor Immunotherapy. Front. Immunol. 12, 672356. 10.3389/fimmu.2021.672356 33936118 PMC8085349

