# Supplementary material for: Identify the Prognostic and Immune Profile of VSIR in the Tumor Microenvironment: A Pan-Cancer Analysis
Source: Front Cell Dev Biol. 2022 Apr 12;10:821649. doi: 10.3389/fcell.2022.821649 (PMC9039624; doi:10.3389/fcell.2022.821649)
Supplement: Supplementary file 5 [file DataSheet1.docx]

**Supplementary Figures**

**
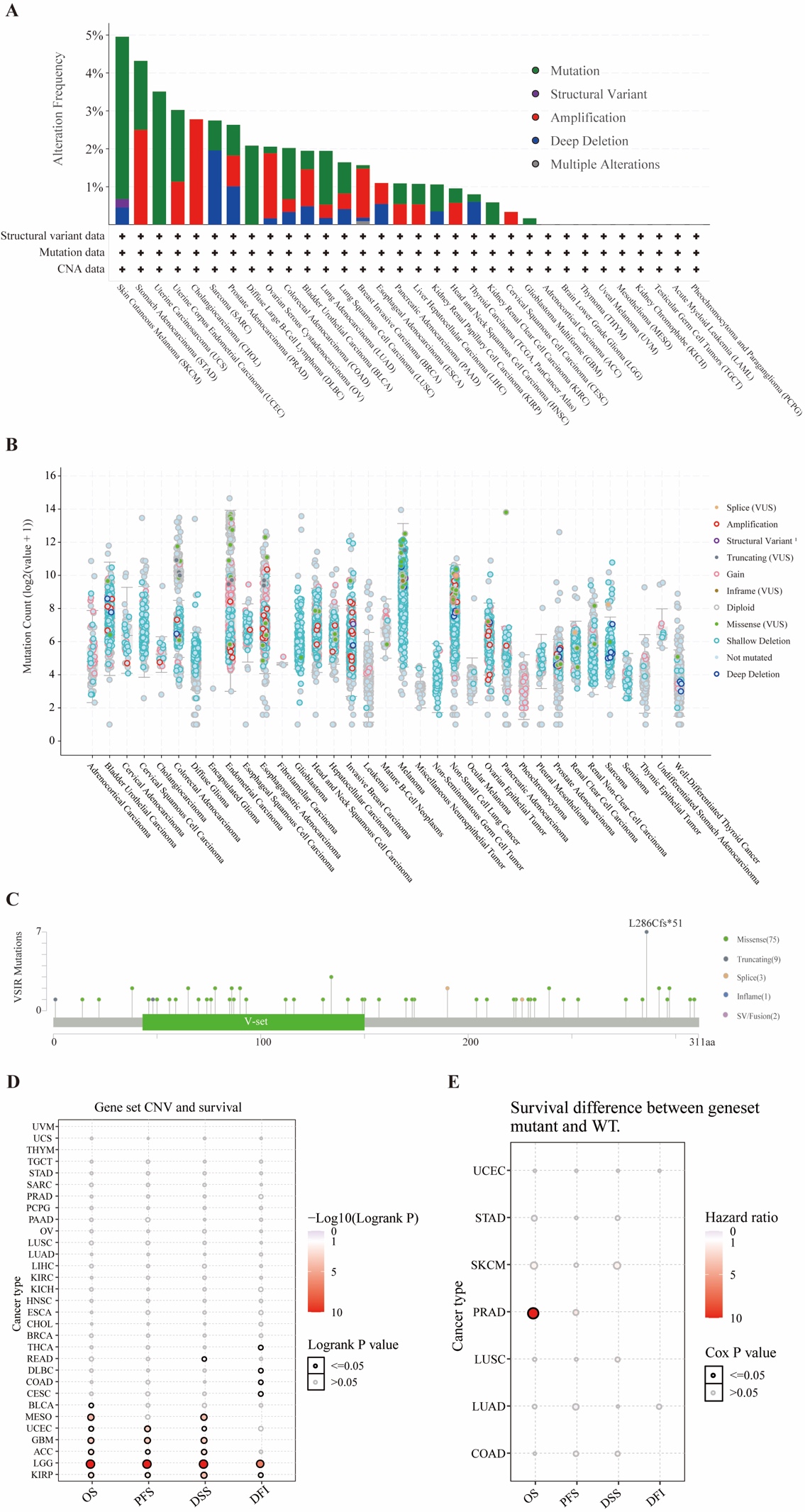
**

**Figure S1. Mutation characteristics of VSIR in pan-cancer. Mutation frequency (A) and entire mutation count (B) of VSIR in pan-cancer. Mutation profile of VSIR across protein domains (C). The relationship between VSIR set CNV and survival (D). Survival difference between VSIR mutant and WT (E). ACC: Adrenocortical carcinoma; BRCA: Breast invasive carcinoma; CESC: Cervical squamous cell carcinoma and endocervical adenocarcinoma; CHOL: Cholangiocarcinoma; COAD: Colon adenocarcinoma; ESCA: Esophageal carcinoma; GBM: Glioblastoma; HNSCC: Head and neck squamous cell carcinomas; KICH: Kidney chromophobe; KIRC: Kidney renal clear cell carcinoma; KIRP: Kidney renal papillary cell carcinoma; LAML: Acute myeloid leukemia; LGG: Low-grade glioma; LIHC: Liver hepatocellular carcinoma; LUSC: Lung squamous cell carcinoma; O.V.: Ovarian Cancer; PAAD: Pancreatic adenocarcinoma; PCPG: Pheochromocytoma, and paraganglioma; PRAD: Prostate adenocarcinoma; READ: Rectum adenocarcinoma; SARC: Sarcoma; SKCM: Skin cutaneous melanoma; STAD: Stomach adenocarcinoma; TGCT: Testicular germ cell tumors; THCA: Thyroid carcinoma; THYM: Thymoma; UCS: Uterine carcinosarcoma; UVM: Uveal melanoma; LSCC: laryngeal squamous cell carcinoma; UTUC: upper tract urothelial cancer; PSCC: Penile squamous cell carcinoma.**

**
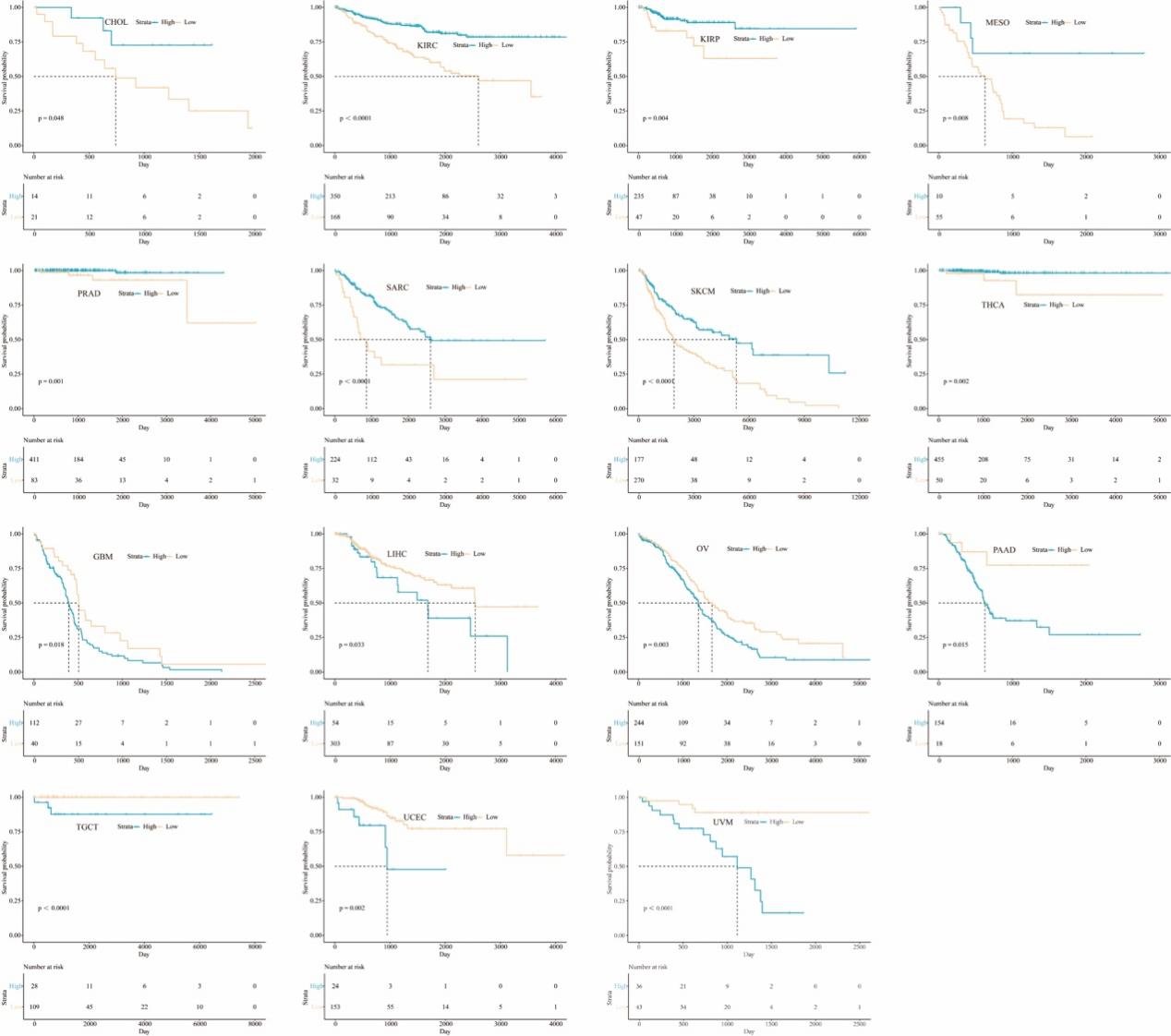
**

**Figure S2. KM displayed the predictive value of VSIR on DSS in pan-cancer.**

**
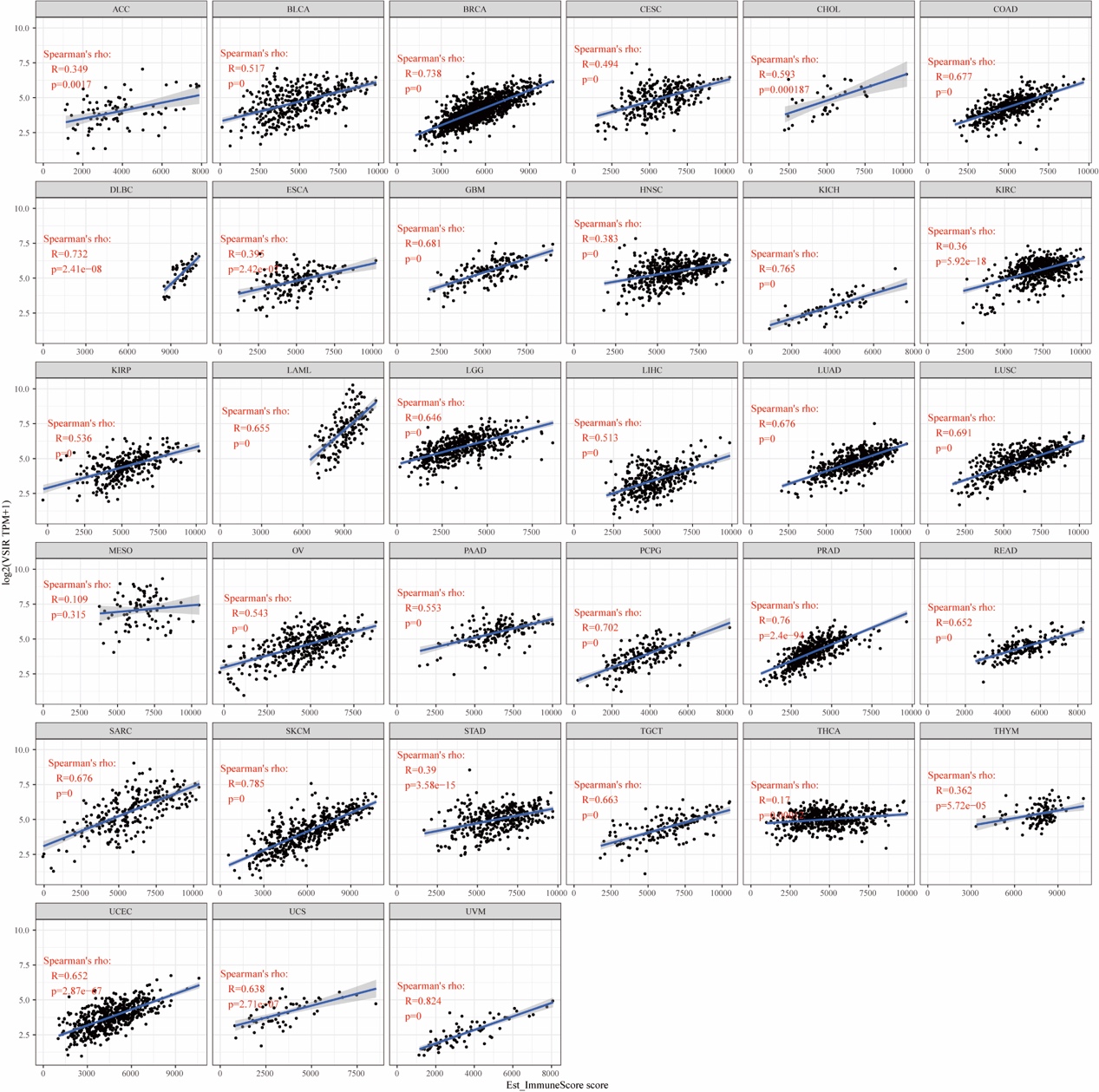
**

**Figure S3. Relationship between immune score and VSIR expression.**

**
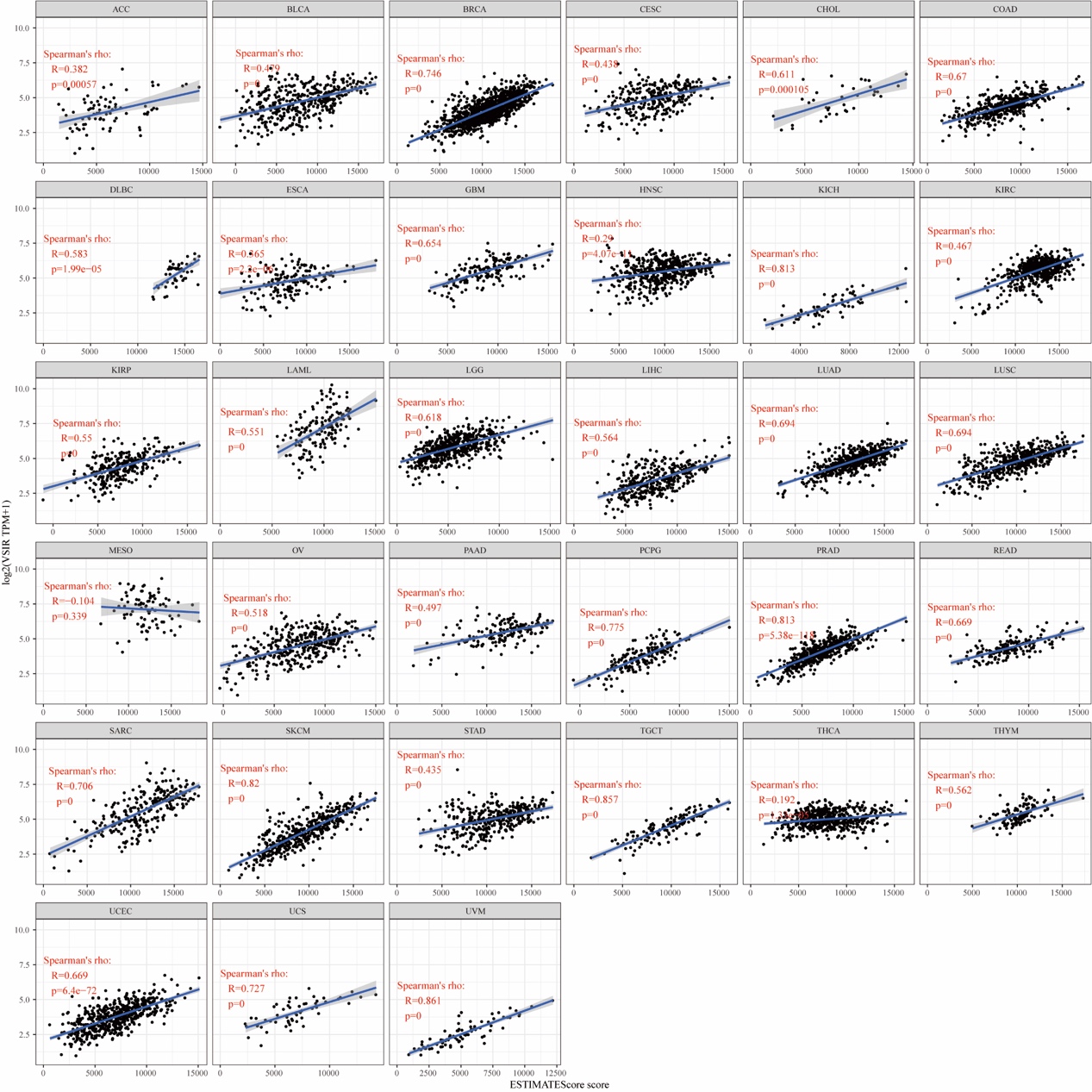
**

**Figure S4. Relationship between estimate score and VSIR expression.**

**
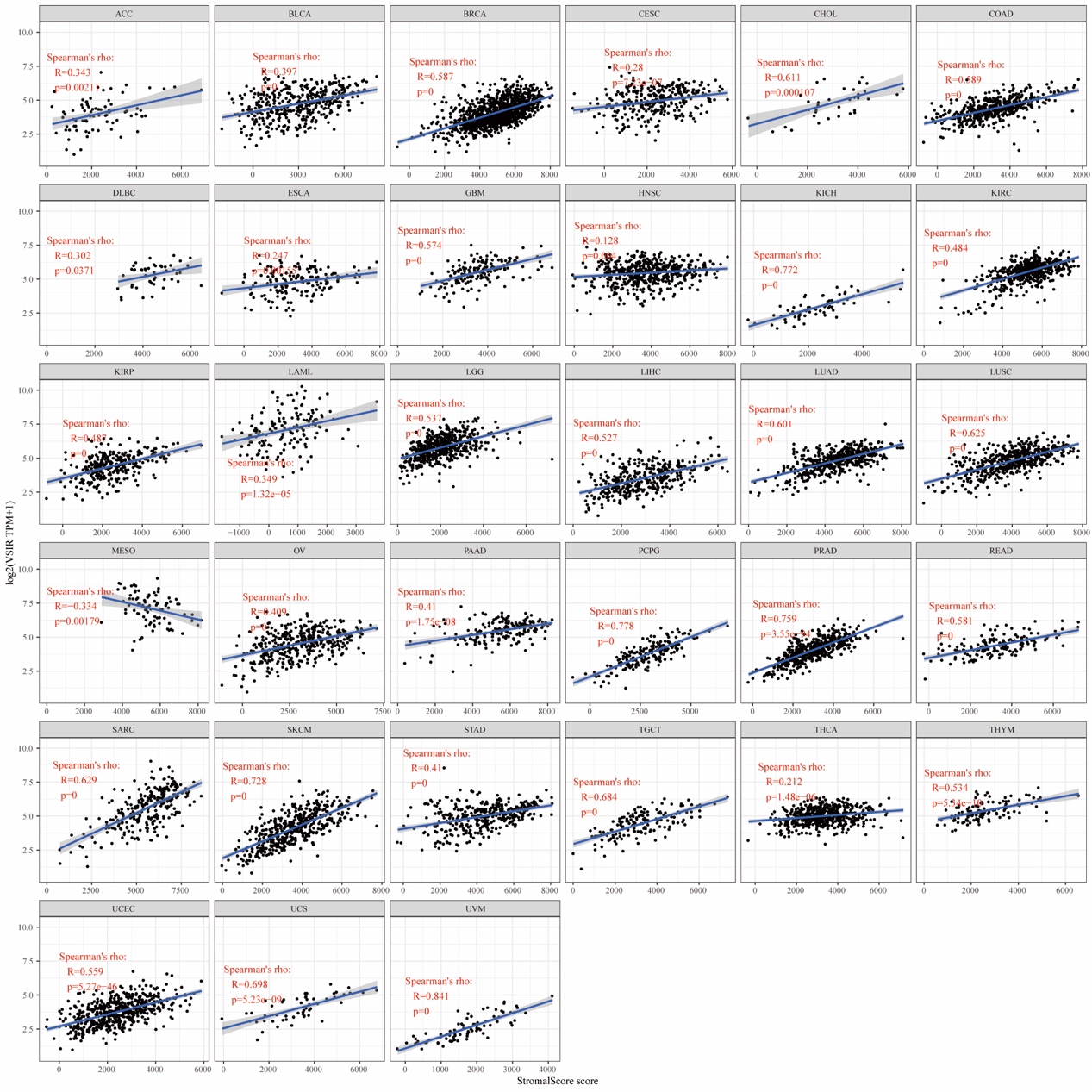
**

**Figure S5. Relationship between stromal score and VSIR expression.**

**
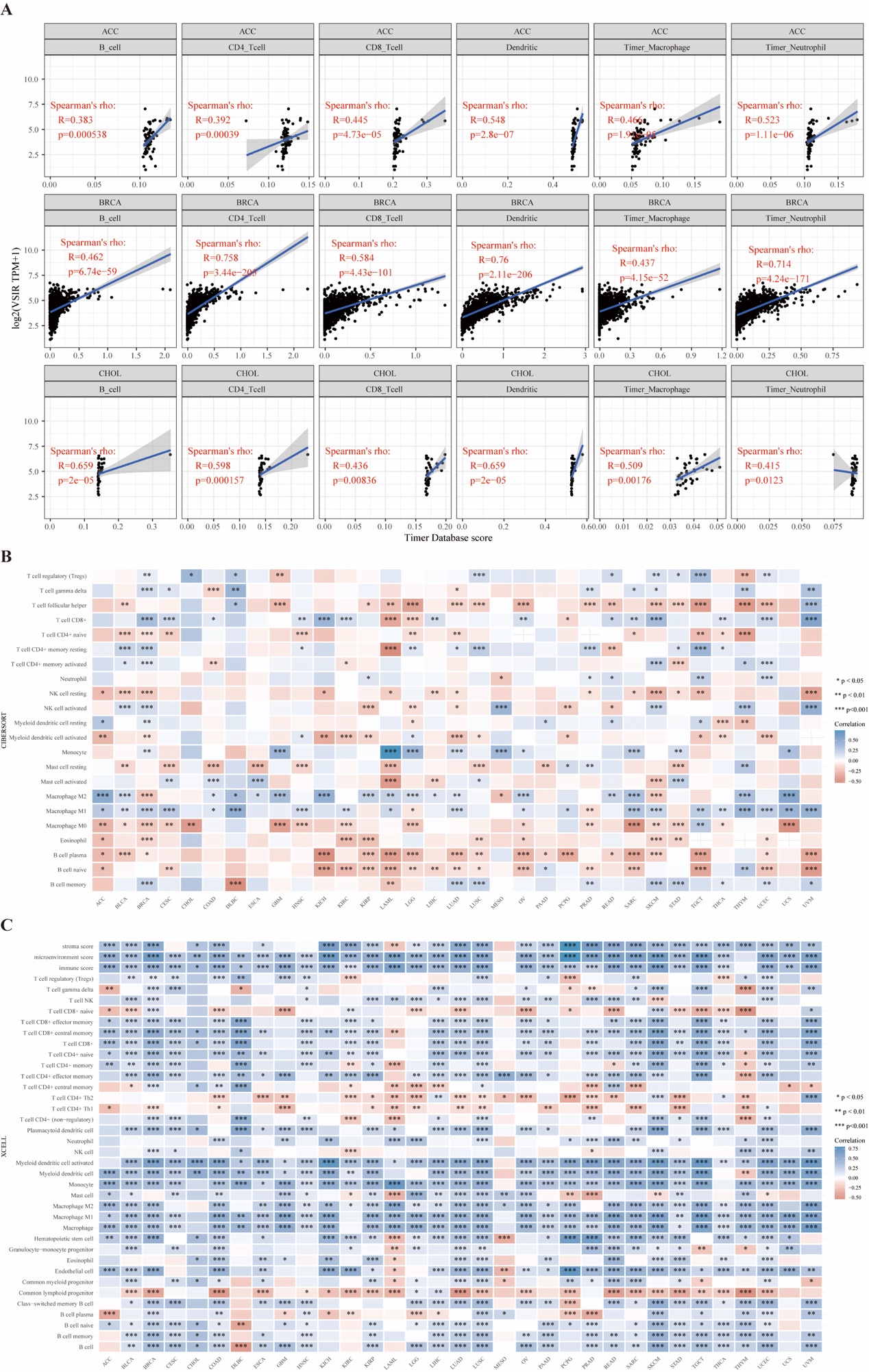
**

**Figure S6. Top three cancers that most positively correlated with immune infiltrates in the TME (A). Correlation between VSIR and infiltrated inflammatory cells analyzed by the immunedeconv algorithm. Immune cell infiltration was analyzed by the CIBERSORT (B) and xCell (C) algorithms. *p< 0.05, **p < 0.01, ***p < 0.001.**

**
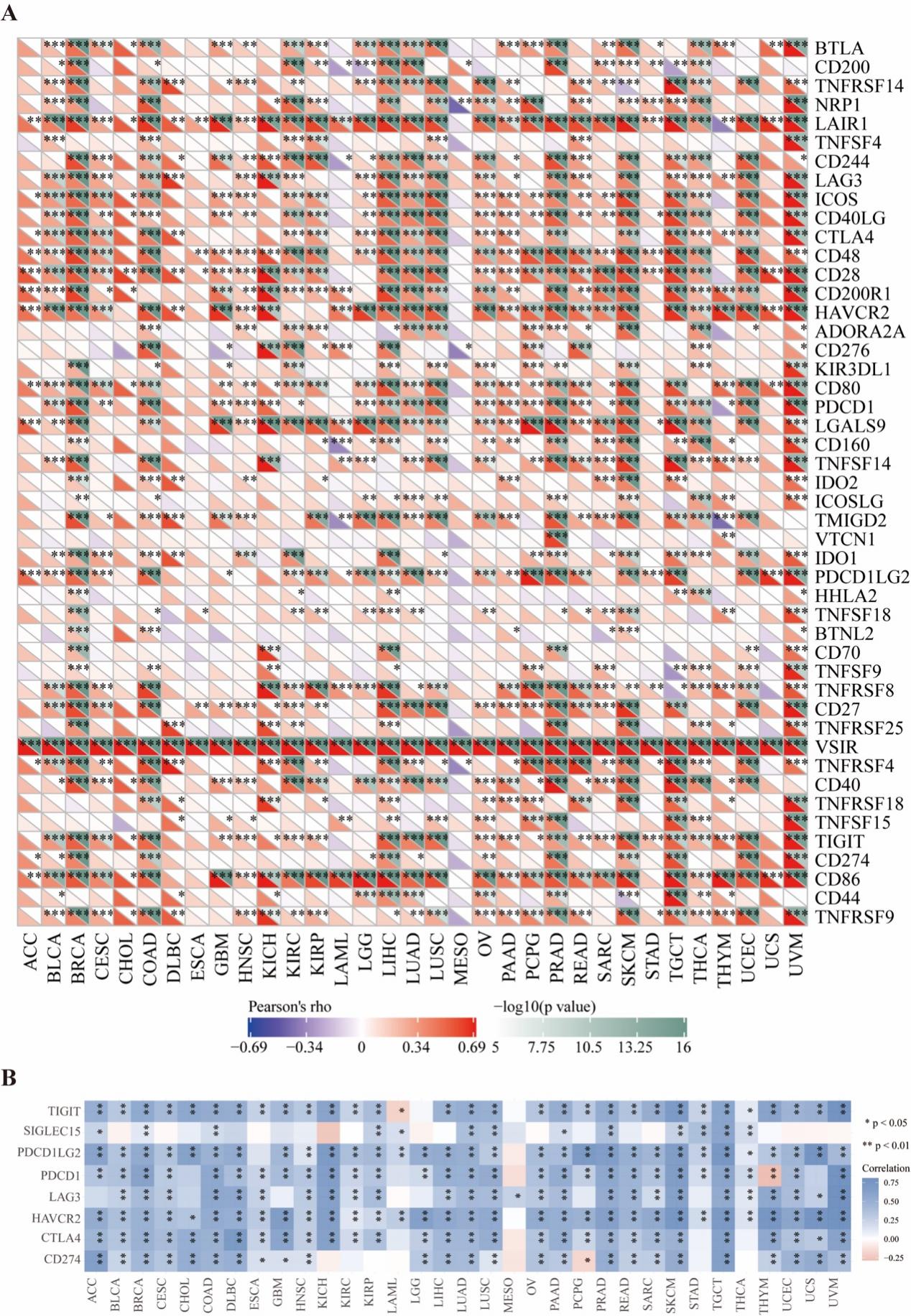
**

**Figure S7. Relationship between VSIR levels and immune checkpoints. The correlation between VSIR expression and other immune checkpoints is based on the sangerbox (A) and immunedeconv (B) algorithms. p< 0.05, **p < 0.01, ***p < 0.001.**

**
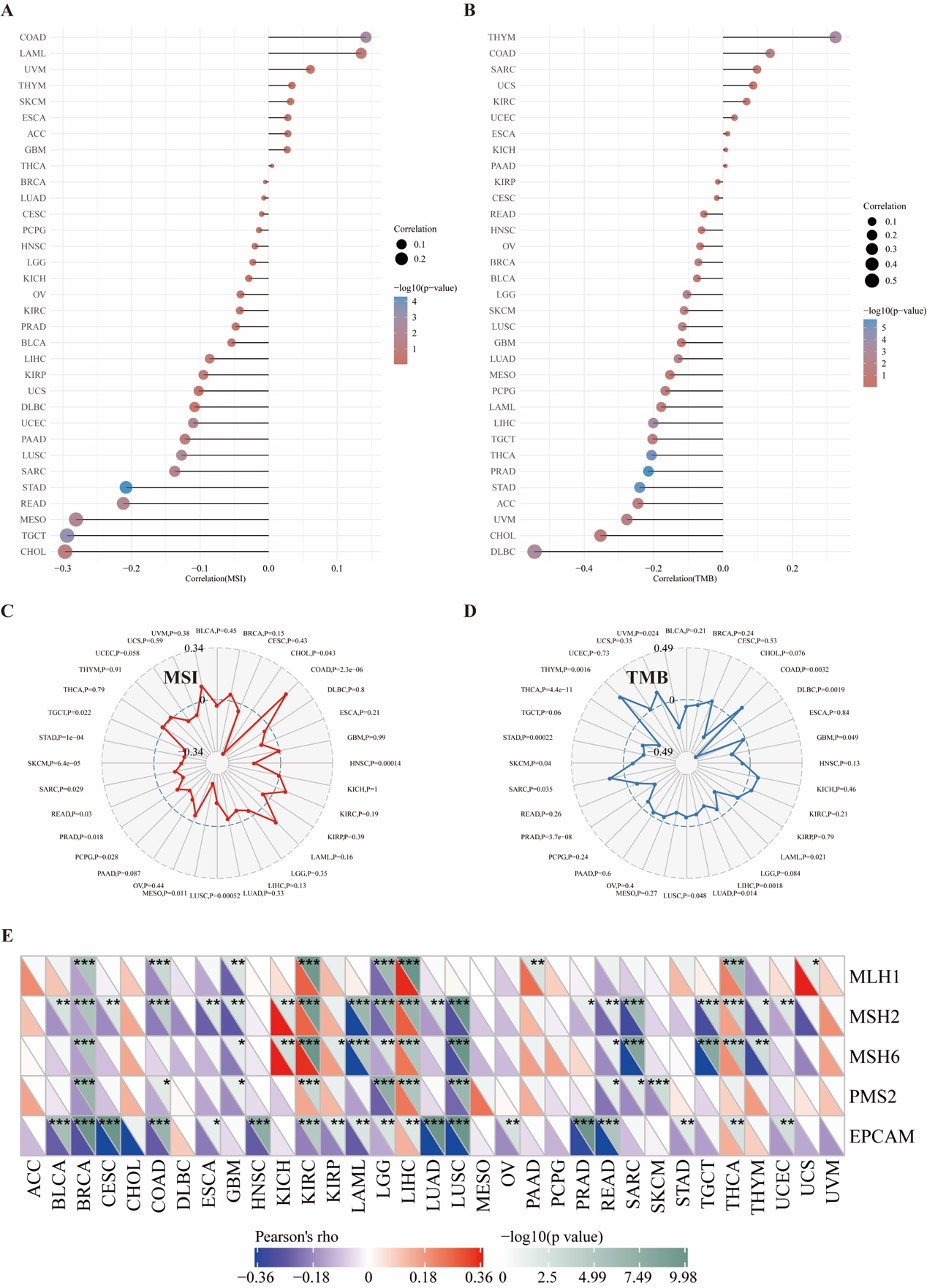
**

**Figure S8. Relationship between VSIR expression and MSI, TMB, and MMRs in pan-cancer. The relationship between VSIR expression and MSI is displayed by the forest plot (A) and radar chart (C). Relationship between VSIR expression and TMB displayed by the forest plot (B) and radar chart (D). Relationship between VSIR expression and MMRs (E). p< 0.05, **p < 0.01, ***p < 0.001.**

**
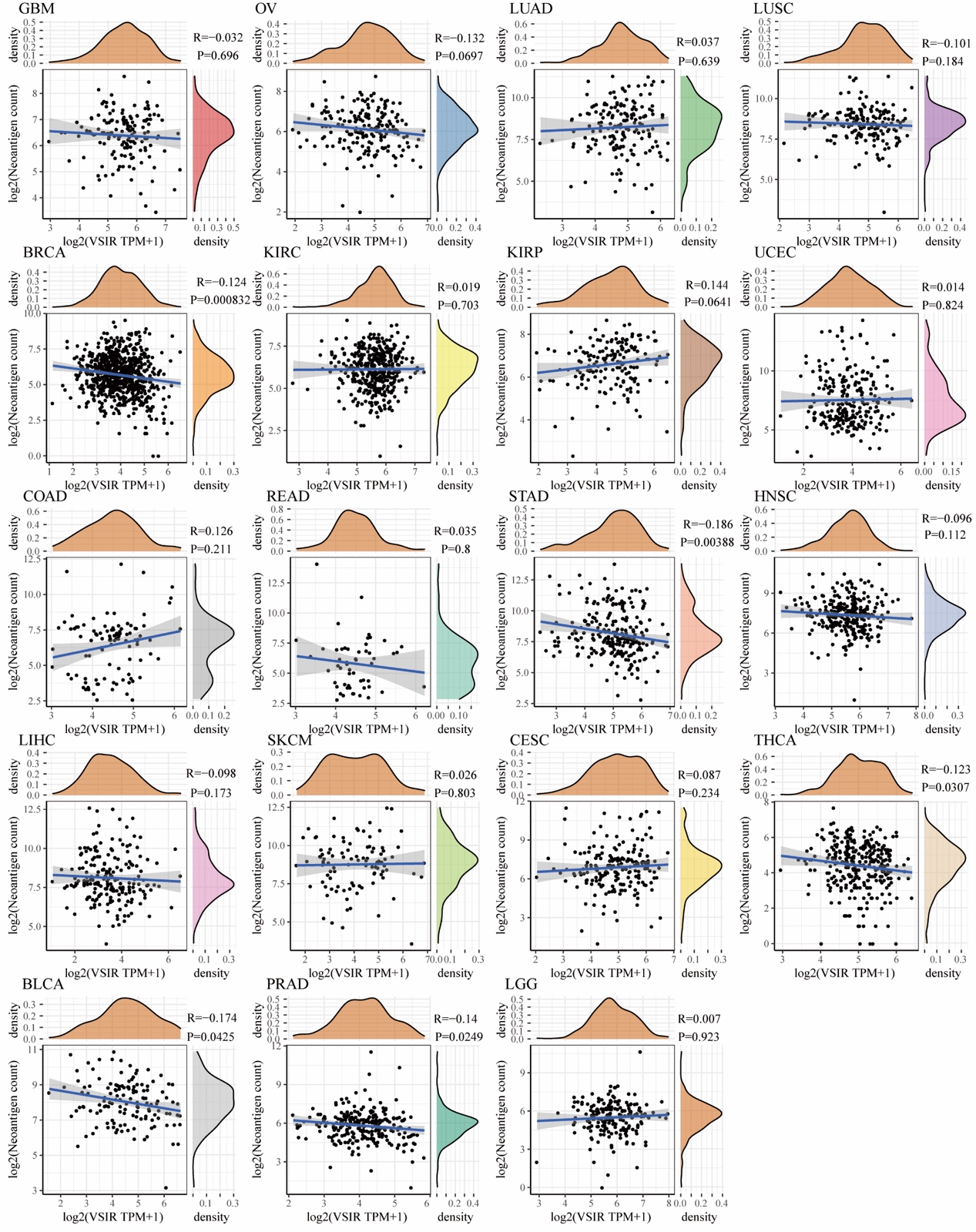
**

**Figure S9. Relationship between VSIR levels and neoantigens in pan-cancer.**

**
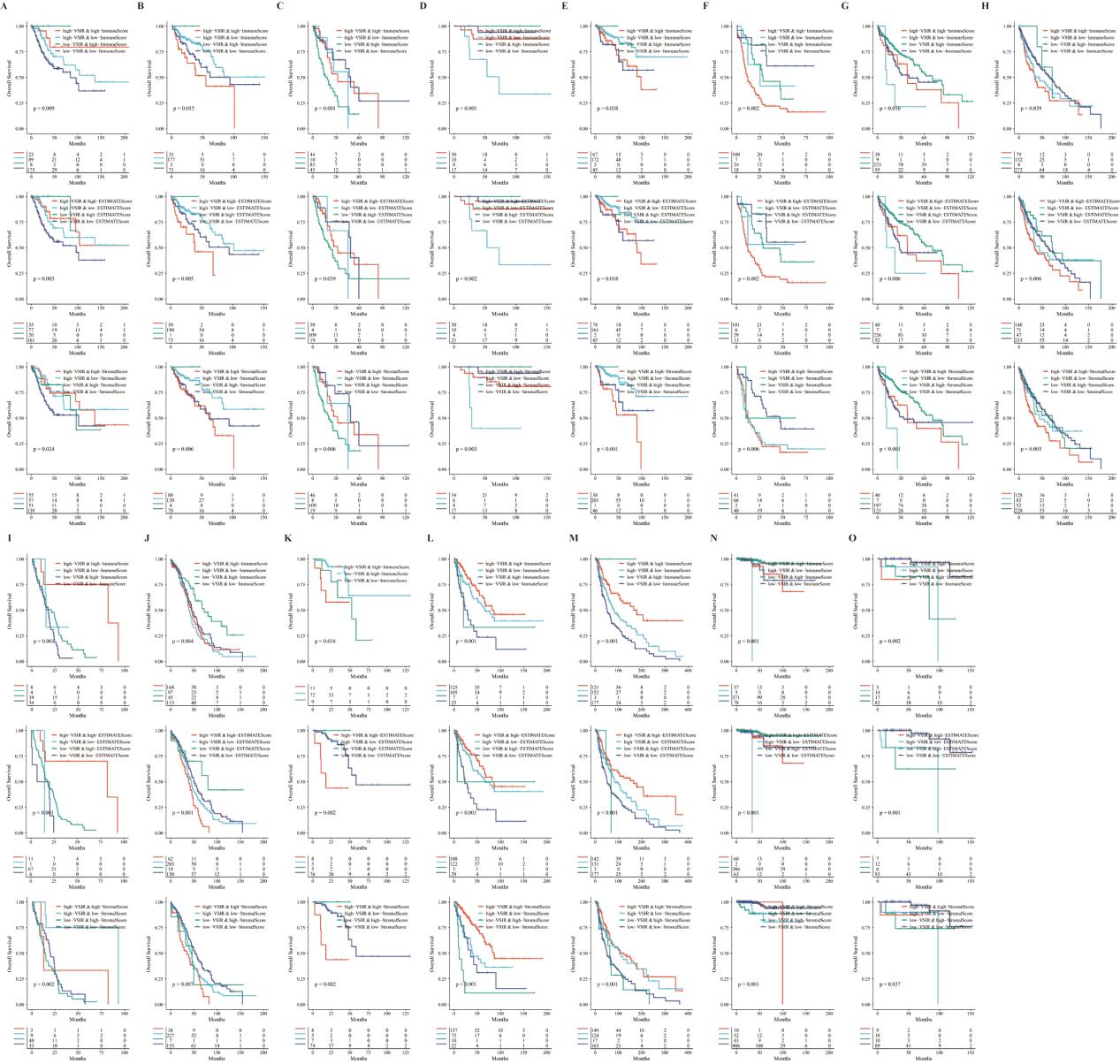
**

**Figure S10. Role of VSIR in pan-cancer regarding VSIR expression, prognostic role, and association with tumor immunity in CESC (A), COAD (B), ESCA (C), KICH (D), KIRP (E), LAML (F), LIHC (G), LUSC (H), MESO (I), OV (J), READ (K), SARC (L), SKCM (M), THCA (N), and THYM (O).**

**
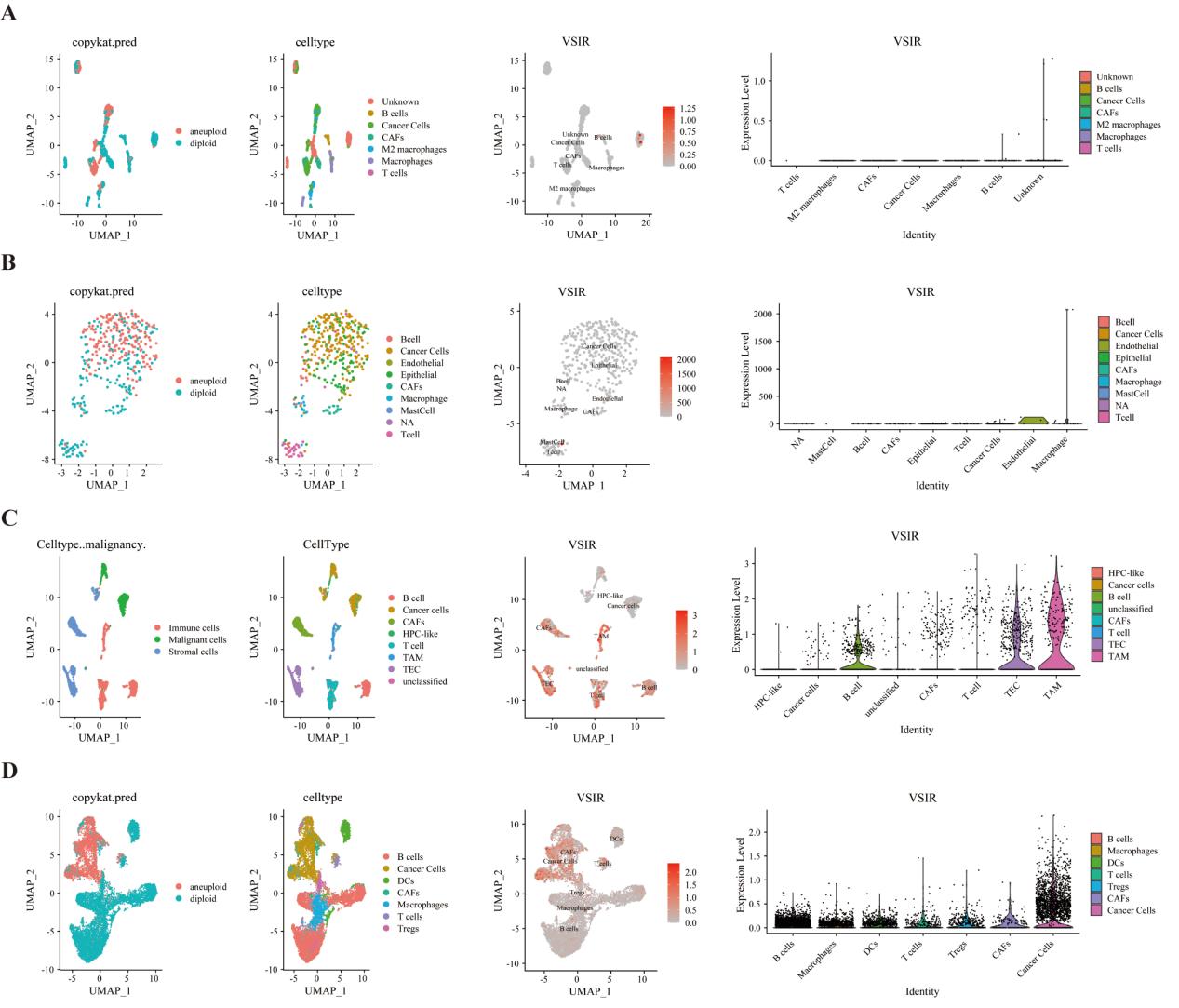
**

**Figure S11. Single-cell sequencing analysis of VSIR in the TME. The relationship between VSIR levels and cancer cells and stromal cells in BRCA (A), COAD (B), LIHC (C), and STAD (D).**

**Supplementary tables**

**Table S1. Detailed information of survival difference between gene set CNV and wide type in pan-cancer.**

**Table S2. Detailed information of survival difference between gene set mutant (deleterious) and wide type in pan-cancer.**

**Table S3. The correlation between VSIR expression and the sensitivity of GDSC drugs (top 30) in pan-cancer.**

**Table S4. The correlation between VSIR expression and the sensitivity of CTRP drugs (top 30) in pan-cancer.**
